# Breast-Gynaecological & Immuno-Oncology International Cancer Conference (BGICC) Consensus and Recommendations for the Management of Triple-Negative Breast Cancer

**DOI:** 10.3390/cancers13092262

**Published:** 2021-05-08

**Authors:** Hesham Elghazaly, Hope S. Rugo, Hamdy A. Azim, Sandra M. Swain, Banu Arun, Matti Aapro, Edith A. Perez, Benjamin O. Anderson, Frederique Penault-Llorca, Pierfranco Conte, Nagi S. El Saghir, Cheng-Har Yip, Marwan Ghosn, Philip Poortmans, Mohamed A. Shehata, Armando E. Giuliano, Jessica W. T. Leung, Valentina Guarneri, Joseph Gligorov, Bahadir M. Gulluoglu, Hany Abdel Aziz, Mona Frolova, Mohamed Sabry, Charles M. Balch, Roberto Orecchia, Heba M. El-Zawahry, Sana Al-Sukhun, Khaled Abdel Karim, Alaa Kandil, Ruslan M. Paltuev, Meteb Foheidi, Mohamed El-Shinawi, Manal ElMahdy, Omalkhair Abulkhair, Wentao Yang, Adel T. Aref, Joaira Bakkach, Nermean Bahie Eldin, Hagar Elghazawy

**Affiliations:** 1 Clinical Oncology Department, Faculty of Medicine, Ain Shams University, Cairo 11566, Egypt; hanyhns@yahoo.com (H.A.A.); mohammad.sabry@med.asu.edu.eg (M.S.); khalidakm@med.asu.edu.eg (K.A.K.); Nermean.mostafa@med.asu.edu.eg (N.B.E.); dr.hagar.elghazawy@med.asu.edu.eg (H.E.); 2 Department of Medicine, University of California San Francisco Comprehensive Cancer Center, San Francisco, CA 94158, USA; 3 Clinical Oncology Department, Kasr Alainy School of Medicine, Cairo University, Giza 12613, Egypt; azimonc@cairocure.com (H.A.A.); hebaelzaw@yahoo.co.uk (H.M.E.-Z.); 4 Lombardi Comprehensive Cancer Center, Georgetown University Medical Center, MedStar Health, Washington, DC 20007, USA; sms248@georgetown.edu; 5 Department of Breast Medical Oncology, The University of Texas MD Anderson Cancer Center, Houston, TX 77030, USA; barun@mdanderson.org; 6 Breast Center, Clinique de Genolier, 1272 Genolier, Switzerland; maapro@genolier.net; 7 Department of Hematology & Oncology, Mayo Clinic, Jacksonville, FL 32224, USA; perez.edith@mayo.edu; 8 Breast Health Global Initiative, Fred Hutchinson Cancer Research Center, University of Washington, Seattle, WA 98195, USA; banderso@fredhutch.org; 9 Department of Pathology, Clermont Auvergne University, INSERM U1240 “Molecular Imaging and Theranostic Strategies”, Center Jean Perrin, Montalembert, 63000 Clermont-Ferrand, France; Frederique.PENAULT-LLORCA@clermont.unicancer.fr; 10 Department of Surgery, Oncology and Gastroenterology, University of Padova, Istituto Oncologico Veneto IOV IRCCS, 35128 Padova, Italy; pierfranco.conte@unipd.it (P.C.); valentina.guarneri@unipd.it (V.G.); 11 Department of Internal Medicine, Division of Hematology Oncology, American University of Beirut Medical Center, Beirut 1107 2020, Lebanon; ns23@aub.edu.lb; 12 Subang Jaya Medical Centre, Kuala Lumpur 47500, Malaysia; chenghar.yip@gmail.com; 13 Hematology and Oncology Department, Saint Joseph University, Beirut 1104 2020, Lebanon; marwanghosnmd@yahoo.com; 14 Iridium Kankernetwerk and Faculty of Medicine and Health Sciences, University of Antwerp, 2610 Wilrijk-Antwerp, Belgium; philip.poortmans@telenet.be; 15 Clinical oncology Department, Menoufia University, Shebin Elkom 51132, Egypt; Mohamed.Shehata@med.menofia.edu.eg; 16 Department of Surgery, Surgical Oncology Division, Cedars-Sinai Medical Center, Los Angeles, CA 90048, USA; armando.giuliano@cshs.org; 17 Department of Breast Imaging, Division of Diagnostic Imaging, The University of Texas MD Anderson Cancer Center, Houston, TX 77030, USA; jwleung@mdanderson.org; 18 Institut Universitaire de Cancérologie AP-HP. Sorbonne Université, INSERM U938, 75013 Paris, France; joseph.gligorov@aphp.fr; 19 Breast & Endocrine Surgery Unit, Marmara University School of Medicine, University Hospital, Istanbul 34722, Turkey; bmgulluoglu@marmara.edu.tr; 20 Federal State Budgetary Institution “NN Blokhin National Medical Research Center of Oncology” of the Ministry of Health of the Russian Federation, 127994 Moscow, Russia; drfrolova@yandex.ru; 21 Surgical Oncology Department, The University of Texas MD Anderson Cancer Center, Houston, TX 77030, USA; cmbalch@mdanderson.org; 22 Scientific Directorate, IRCCS European Institute of Oncology (IEO), and University of Milan, 20122 Milan, Italy; roberto.orecchia@ieo.it; 23 Al Hyatt Oncology Practice, Amman 11183, Jordan; salsukhun@yahoo.com; 24 Department of Clinical Oncology, Alexandria School of Medicine, Alexandria 21131, Egypt; alaakandil@alexmed.edu.eg; 25 Russian Association of Oncological Mammology, Department of Breast Tumours of Federal State Budgetary Institution “Petrov Research Institute of Oncology”, 197758 Saint Petersburg, Russia; paltuev@mail.ru; 26 College of Medicine, King Saud Bin Abdulaziz University for Health Sciences, Adult Medical Oncology, Princess Noorah Oncology Center, King Abdulaziz Medical City, Ministry of National Guard Health Affairs-Western Region, Jeddah 22384, Saudi Arabia; FoheidiME@ngha.med.sa; 27 Department of General Surgery, Faculty of Medicine, Ain Shams University, Cairo 11566, Egypt; mohamedshinawi@hotmail.com; 28 Vice President of Galala University, Galala University, Suez 435611, Egypt; 29 Department of Pathology, Ain shams University, Cairo 11566, Egypt; manalelmahdy@med.asu.edu.eg; 30 Oncology Department, Alfaisal university, Alhabib Hospital, Riyad 11533, Saudi Arabia; omalkhair.abulkhair@drsulaimanalhabib.com; 31 Department of Pathology, Fudan University Shanghai Cancer Center, Shanghai 200032, China; yangwt2000@163.com; 32 The School of Public Health, University of Adelaide, Adelaide 5005, Australia; adel.aref@adelaide.edu.au; 33 Biomedical Genomics & Oncogenetics Research Laboratory, Faculty of Sciences and Techniques of Tangier, Abdel Malek Essaadi University, Tangier 90000, Morocco; j.bakkach@uae.ac.ma

**Keywords:** triple-negative breast cancer, consensus, immunotherapy, BRCA mutations, platinum

## Abstract

**Simple Summary:**

Despite the impressive progress in the treatment of triple-negative breast cancer (TNBC), oncologists still face several provocative clinical scenarios in daily practice where clear evidence-based recommendations are lacking, and expert opinion is of utmost importance. In an attempt to seek guidance for these controversial topics in TNBC management, a consensus recommendations session for TNBC was held during the 12th round of the Breast-Gynaecological & Immuno-oncology International Cancer Conference (BGICC) Egypt, 2020. This special session convened a multidisciplinary committee of 35 panellists who specialize in breast cancer care from 13 countries. The consensus covered all the aspects of TNBC management starting from defining TNBC to the management of metastatic disease and highlighted the rapidly evolving landscape in this field. Consensus was reached in 70% of the statements (35/50). In addition, areas of warranted research were identified to guide future prospective clinical trials.

**Abstract:**

*Background*: The management of patients with triple-negative breast cancer (TNBC) is challenging with several controversies and unmet needs. During the 12th Breast-Gynaecological & Immuno-oncology International Cancer Conference (BGICC) Egypt, 2020, a panel of 35 breast cancer experts from 13 countries voted on consensus guidelines for the clinical management of TNBC. The consensus was subsequently updated based on the most recent data evolved lately. *Methods*: A consensus conference approach adapted from the American Society of Clinical Oncology (ASCO) was utilized. The panellists voted anonymously on each question, and a consensus was achieved when ≥75% of voters selected an answer. The final consensus was later circulated to the panellists for critical revision of important intellectual content. *Results and conclusion*: These recommendations represent the available clinical evidence and expert opinion when evidence is scarce. The percentage of the consensus votes, levels of evidence and grades of recommendation are presented for each statement. The consensus covered all the aspects of TNBC management starting from defining TNBC to the management of metastatic disease and highlighted the rapidly evolving landscape in this field. Consensus was reached in 70% of the statements (35/50). In addition, areas of warranted research were identified to guide future prospective clinical trials.

## 1. Introduction

Despite the impressive progress in the treatment of triple-negative breast cancer (TNBC), oncologists still face several provocative clinical scenarios in daily practice where clear evidence-based recommendations are lacking. Expert opinion is of utmost importance for guiding management in these controversial situations. In addition, not all countries have equal access to therapeutic and diagnostic resources. In an attempt to seek guidance for these controversial topics in TNBC management, a consensus recommendations session for TNBC was held during the 12th round of the Breast-Gynaecological & Immuno-oncology International Cancer Conference (BGICC) Egypt, January 2020, with over 3500 participants. This special session convened a multidisciplinary committee of 35 panellists who specialize in breast cancer care (medical, surgical and radiation oncology, pathology and radiology) from 13 countries. The objective of this consensus process was to establish clinical recommendations for selected decisions and treatment sequencing to manage patients with TNBC, distinguishing appropriate approaches for routine standard of care, versus those where knowledge gaps warrant additional evidence, which may direct the design of future clinical trials.

## 2. Materials and Methods

The recommendation statements within the task force were evaluated through a consensus conference approach adapted from the American Society of Clinical Oncology (ASCO) [1], in which the questions and statements were proposed by a steering group and then reviewed and adapted by the whole panel before the BGICC meeting. During the consensus meeting, the panellists (Table 1) were presented a written case and then voted anonymously on multiple-choice statements to select what each thought to be the best management decision. A consensus was defined a priori as ≥75% of voters indicating concordance on a given answer. The panellists were asked to vote (1) yes, if they agreed with the addressed statements; (2) no, if they disagreed with the statement; (3) other options, in some questions; (4) abstain, if they had either insufficient expertise with the specific statement or had a conflict of interest that could influence their vote. The consensus agreement or disagreement rate was calculated without including the abstaining votes in the denominator, in order to avoid affecting the overall rates in questions with mutually exclusive choices. Discussions and comments of the panellists were documented to be considered in the results. Later after this meeting, updated data for some immunotherapy studies in TNBC were presented at the 2020 ASCO, European Society for Medical Oncology (ESMO) and San Antonio Breast Cancer Symposium (SABCS), reflecting the rapid dynamics in this field, where relevant statements were developed, revised by the panel and voted upon through emails. The final consensus manuscript was later circulated to the panel members for critical revision of important intellectual content.

Recommendation statements were developed based on the current literature using a modified GRADE (Grading of Recommendations, Assessment, Development, and Evaluations) methodology. When necessary, expert opinion supplemented the evidence [2] (Table 2). The quality of evidence underlying each recommendation statement was categorized from I to V. Levels of evidence were assigned to studies based on the methodological quality of their design, validity and applicability to patient care [2] (Table 3).

## 3. Results

The results of the voting, levels of evidence and grades of recommendation are presented for the consensus statements. Topics which had a voting percentage of 75% or more were considered as “consensus reached”. The consensus was reached in 70% of the statements (35/50). In addition, areas of future research were identified to guide future prospective clinical trials. The discussion on each of the voting topics and the evidence behind are presented in the following section.

## 4. Discussion

### 4.1. Definition of TNBC and Pathology Evaluation

#### 4.1.1. TNBC Definition

Triple-negative breast cancer (TNBC) is a heterogeneous breast cancer (BC) subtype that lacks the expression of oestrogen receptor (ER) and progesterone receptor (PR) and also does not overexpress or show gene amplification of the human epidermal receptor 2 (HER2) oncogene [3]. However, in the literature, there is variability in the threshold for defining ER, PR negativity, including ≤10%, ≤5% or <1% of tumour cells that express hormonal receptors [4,5]. *For this statement, 85.5% of panellists accepted the threshold for ER and PR negativity at <1%. This vote was supported by the updated American Society of Clinical Oncology/College of American Pathologists (ASCO/CAP) guidelines [6]. Nevertheless, the panel completely acknowledged the growing body of evidence, which strongly suggests several biological similarities between TNBC and HER2− tumours with low ER positivity (1–10%).*

#### 4.1.2. HER2 Negative (HER2−) and ER/PR Expression 1–10%

Estrogen receptor low-positive tumours (ERLP), defined as 1–10% ER positivity by immunohisto-chemistry (IHC), are relatively uncommon, accounting for 2–6% of all BCs [7,8]. Although the updated ASCO/CAP 2020 guidelines [6] still consider these tumours as ER-positive tumours, they recommended classifying them differently as ERLP. Emerging data strongly suggest that patients with ERLP tumours do not significantly benefit from adjuvant endocrine therapy [9] and have worse overall survival compared to those with ≥10% ER-positive tumours [10,11,12]. A study including 3055 patients from MD Anderson Cancer Center demonstrated that among stage II–III patients with ER ≥10% tumours, but not those with ER 1–9%, adjuvant endocrine therapy was significantly associated with longer time to recurrence (Hazard Ratio (HR) = 0.24, *p* < 0.001 versus (vs.) HR = 0.88, *p* = 0.67 in ER 1–9%) and overall survival (OS) (HR = 0.32, *p* < 0.001 vs. HR = 0.65, *p* = 0.25 in ER 1–9%) [9]. Importantly ERLP and TNBC share several biological features, including aggressive natural history, dominance of basal-like intrinsic subtype, high pathological complete response (pCR) rate to neo-adjuvant chemotherapy and poor prognosis [8,10,11,12]. Moreover, the incidence of *BRCA1/2* mutations in patients with ERLP tumours is comparable to that encountered in TNBC patients [13].

The omission of endocrine therapy in this group of patients, so that they are managed similarly as TNBC patients, is still controversial. It is also unclear whether it is a required therapy in the adjuvant setting, metastatic setting, or both. In light of this evidence, the threshold for hormone receptor positivity that predicts the benefit of endocrine therapy has been questioned [14]. In 2015, St. Gallen Consensus reported that ER expression between 1–9% was considered equivocal and that endocrine therapy alone cannot be relied upon as adequate treatment for these patients [15]. *Given the controversy about the benefit of endocrine therapy and the limited available data for ERLP, the panel highlighted that separate studies for this peculiar subgroup are warranted.* *However, although it is recommended to consider BC with HER2− and ER/PR expression between 1 and 10% as hormone-sensitive, 61.5% of the panel agreed that these tumours would be treated clinically as TNBC, being not eligible to receive endocrine therapy as a monotherapy. The rest of the panel opted for keeping ERLP tumours not “non-eligible” for endocrine therapy. However, no consensus was reached in this context and the panel members believed that endocrine therapy has no significant benefit in this subgroup and should not be considered a sole requirement in management. Moreover, in the 2021 St. Gallen Consensus, there was still no clear recommendation regarding the appropriate threshold to recommend endocrine therapy [16].*

#### 4.1.3. Germline *BRCA* Mutation Testing in Early TNBC Patients

A meta-analysis including 46,870 BC patients concluded that patients with germline *BRCA1 (gBRCA1)* mutations were 3- and 9-fold more likely to have TNBC compared to *gBRCA2* carriers and non-carriers, respectively [17]. On the other hand, the prevalence of *gBRCA* mutations in TNBC patients varies according to selection criteria. A study including 2,733 young BC patients (<40 years) showed a 24% prevalence rate of *gBRCA* mutations among TNBC patients (136/558) [18]. Another study including 802 TNBC patients without a family history of breast or ovarian cancer showed that the prevalence of *gBRCA* mutations was 16% [19]. Overall, data from the literature indicate that 9–32% of unselected TNBCs were *gBRCA* mutation carriers [20]. This was in line with the National Comprehensive Cancer Network (NCCN) guidelines for genetic/familial high-risk assessment (version 2, 2021), which recommended *gBRCA* testing for TNBC patients diagnosed at age ≤60 years irrespective of familial background and for BC patients diagnosed at any age with a strong family history [21]. *Regarding which TNBC patients are to be tested for gBRCA mutations at initial diagnosis of early BC in order to offer genetic counselling and risk-reduction measures, 67% of the panel believed that testing should be routinely performed for early TNBC patients either diagnosed at age ≤60 years or those of any age with a strong family history. However, 33% of the panel agreed that testing should be requested for all patients with TNBC.*

#### 4.1.4. Androgen Receptor (AR) Immunohistochemistry (IHC) Reporting in TNBC

Although many research studies investigated different androgen receptor (AR) inhibitors in the treatment of AR+ metastatic TNBC (mTNBC), such therapeutic strategies still show only modest clinical outcomes [22,23,24]. In 2011, Lehmann et al. [25] subcategorized TNBC into six subtypes, including the luminal androgen receptor (LAR) subtype, which is characterized by the overexpression of AR and its downstream effects. It is reported that AR is overexpressed in 10–41% of TNBCs [25,26]. However, the clinical and prognostic value of AR expression is still controversial [27], whereas few phase II studies have evaluated the clinical value of AR blockade in AR+ mTNBC, showing modest results [28]. Results from ongoing studies evaluating different AR blocking agents in early and mTNBC are still awaited [29,30]. Those studies used 10% as a cut off for AR positivity by IHC, although this is an area for further research. *After discussion, 77% of the panel endorsed that AR testing can not guide treatment decision at the time being, pending the presence of mature data for the efficacy and safety of AR blockade in AR+ TNBC. The panel believed that it should be reported for research purposes only.*

#### 4.1.5. Tumour-Infiltrating Lymphocytes (TILs) Reporting in TNBC

The prognostic value of tumour-infiltrating lymphocytes (TILs) in TNBCs has been well documented in numerous clinical studies in different settings [31,32,33]. A pooled data analysis has recently confirmed the correlation between TILs and disease-free and overall survival [34]. A high infiltration by TILs was also predictive of response in patients with early TNBC treated with neo-adjuvant therapy (NAT) [35,36], being correlated with better pCR [37]. However, no trials discussed the impact of TILs on guiding the treatment decision until now, which is a reflection that there is still a need for more evidence to support its predictive value and impact on the clinical decision. *There was a split voting regarding the importance of reporting TILs. Forty-four percent of the panel believed that reporting TILs should be restricted to research purposes only, albeit 34% of the panellists agreed on the routine reporting of TILs for its prognostic value. A minority of the panel (22%) felt that routine reporting of TILs may help the clinicians in decisions related to escalating or de-escalating chemotherapy regimens during the neo/adjuvant or metastatic setting. The overall conclusion was still not in favour of the routine use of TILs due to a lack of solid evidence. This is also consistent with the 2021 St. Gallen Consensus [16].*

#### 4.1.6. TILs Evaluation in Tumour Pathology Samples

In early studies, TILs reported on tumour pathology samples were split into two types: (1) lymphocytes, which are in direct contact with malignant cells and are referred to as intra-tumoural TILs, and (2) those located within the tumour stroma yet not in direct contact with malignant cells, referred to as stromal TILs [38]. Reporting intra-tumoural TILs can be challenging as they are not as abundant as stromal TILs and less reproducible on H&E stained slides, so it would seem more feasible to report TILs according to their presence in the tumour stroma [39]. In 2014, the International TILs Working Group published a recommendation in order to unify the methods of assessment of TILs and explore their clinical relevance, which endorsed using stromal TILs for standard reporting [39]. More recent studies evaluating TILs in TNBC before/after NAT showed that both stromal and intra-tumoural TILs are predictive of pCR, though the presence of stromal TILs seemed to be more significant [40,41].

*When addressing this topic, 48% of the panellists voted for stromal TILs, and 52% of the panellists voted for the importance of reporting both stromal and intra-tumoural TILs. The panel felt that more data are required to come out with a final conclusion regarding the clinical impact of reporting TILs in TNBC and to be prioritized in future research.*

#### 4.1.7. Reporting of Ki-67 in TNBC

Ki-67 is a nuclear protein whose expression has been proposed to be a measure for quantification of cell proliferation in BC [42]. The role of Ki-67 is well established in guiding treatment decisions in luminal BC (although poor inter-pathology validation obscures cross-institutional validity), whereas its role in TNBC is so far poorly defined [43,44]. In a meta-analysis including 7716 early TNBC patients, high Ki-67 expression was significantly associated with poor disease-free survival (DFS) (HR = 1.7, 95% CI = 1.45–2.07) and poor OS (HR = 1.6, 95% CI = 1.27–2.14) [45]. Moreover, another study, including 800 early TNBC patients, suggested that Ki-67 is a nearly independent prognostic and predictive factor for both DFS and OS [46]. This study also suggested that Ki-67 cut-off at 30% may help classify TNBC into two subtypes with different responses and prognosis. In this study, TNBC patients with high Ki-67 showed OS improvement with adjuvant chemotherapy, whereas OS was not improved among TNBC patients with low Ki67. The benefit of adjuvant treatment in TNBC patients with stage I and low Ki-67 was not clear. *Based on these data,* *there was a split among the panel about the role of Ki-67 in TNBC. Thirty six percent of the experts agreed that Ki-67 should not be reported in routine practice as it will not guide treatment tailoring, while 34% advised for Ki-67 reporting in TNBC cases for research purposes only and another 30% believed that it might have a role in guiding treatment decisions acknowledging that high Ki-67 expression correlates with lower survival.* *After discussion, the panel* *came to an agreement confirming that currently, Ki-67 had no pivotal role yet in standard practice in TNBC (in contrast to luminal BC [43]), while there is room for more research in this area.*

#### 4.1.8. Retesting Hormonal and HER2 Receptors in Residual Tumours after Neo-Adjuvant Therapy

The BIG-NABCG 2015 [47] recommended retesting hormonal and HER2 receptors status in the residual tumour after NAT in TNBC, pointing to the causes of discordance. In addition, a recently published meta-analysis strongly supported this approach [48]. In this meta-analysis, the rate of conversion of ER-negative to positive was 18.4%, and HER2-negative to -positive was 2.4%. Tumours with hormone receptors negative and converted to positive tend to achieve better DFS (HR = 0.83) and OS (HR = 0.67), compared to tumours that did not gain the receptors’ positivity. Several hypotheses explained these receptors’ switch, including discordances between core needle biopsy and surgically excised samples, intra-tumour heterogeneity and selective killing of sensitive cancer cells, leaving the more resistant strains. *With these available data, 75% of the panellists agreed to repeat hormonal and HER2 receptors assessment after NAT in TNBC. The panel believes that any positivity or overexpression needs to be assessed to avoid missing any opportunity for endocrine or anti-HER2 therapy in the adjuvant setting.*

#### 4.1.9. Anti-HER2 Targeted Therapy in HER2 Switch-to-Positive after Neo-Adjuvant Therapy

*There was a consensus (92.6%) to offer anti-HER2 therapy in the case of switch of HER2 receptor from negative to overexpressed after NAT. The panel felt that this is an appropriate approach, despite the lack of strong evidence to support this decision.*

### 4.2. Loco-Regional Treatment for TNBC

#### 4.2.1. Mastectomy Versus Conservative Surgery for Early TNBC Cases (cT1−2 N0)

There are conflicting data regarding the impact of TNBC biology on loco-regional recurrence (LRR). Some retrospective studies showed higher LRR rates after breast conservative surgery (BCS), while others did not [49,50,51]. A study based on the Surveillance, Epidemiology and End Results (SEER) database analysed outcomes of BCS compared to mastectomy in a cohort of 14,910 patients diagnosed with T1−2 N0 M0 TNBC (2010–2014). There was a significant survival benefit favoring breast-conserving therapy (BCT) vs. mastectomy, regardless of whether this latter was with radiation therapy or not. The 5-year OS were 88.6%, 83% and 79.6% for BCT, mastectomy only and mastectomy followed by radiation therapy, respectively [52]. A meta-analysis by Wang et al. [53] concluded that BCT could be more beneficial in terms of ipsilateral LRR and distant metastasis to patients with stage I–II TNBC compared to mastectomy, owing to the contribution of the postoperative radiation therapy. Of note, the TNBC subtype increased the risks of both ipsilateral LRR (Relative Risk (RR) = 1.88) and distant metastasis (RR = 2.12) compared to non-TNBC subtypes [53]. *There was a 100% consensus amongst the panellists to reject mastectomy as the preferred option over BCS in these early cases* *based upon the TNBC subtype only.*

#### 4.2.2. Surgical Options for *gBRCA1/2* Mutant Early TNBC (Ipsilateral)

Evidence about the oncological safety of BCS among *gBRCA* mutation carriers comes mainly from retrospective studies, and available data are conflicting [54,55]. Compared to mastectomy, an increased risk for recurrence in *gBRCA* mutation carriers has been observed in patients treated with BCS [56]. A recent meta-analysis had demonstrated that ipsilateral BC recurrence rates in *gBRCA* mutation carriers, at 15 years, were higher in the BCS group than in the mastectomy group (23% vs. 6.4%, respectively). However, the 15-year OS was comparable (83.6% vs. 83.2%, respectively) [57]. At the present time, skin-sparing mastectomy is widely recommended for patients with early-stage BC as it can achieve a radical cure while resolving important cosmetic issues [58]. Patient preference remains an important factor to be considered, although available data suggest that many *gBRCA* mutation carriers, at least in western countries, may prefer to undergo bilateral mastectomies [59]. *In the case of gBRCA1/2 mutant early TNBC, 57% of the panel agreed that the preferred surgical option for the affected breast is mastectomy/skin-sparing mastectomy, while 29% believed that there is no preference for a specific type of surgery and 14% voted for conservative surgery. This challenging split of votes, especially in the absence of randomized controlled trials that directly compare BCS vs. mastectomy in gBRCA mutation carriers, steered the panel to consider the patient’s preference in this concern. This was in line with the latest hereditary BC management guideline [60].*

#### 4.2.3. Risk-Reduction Contra-Lateral Mastectomy for *gBRCA1/2* Mutant Early TNBC

A Dutch multi-center prospective study involving around 6000 invasive BC patients showed that the 10-year cumulative risk of developing contra-lateral BC was 21%, 10.8% and 5% for *gBRCA1*, *gBRCA2* mutation carriers and non-carriers respectively [61]. These results highlighted the need for risk reduction measures for the contra-lateral breast in *gBRCA* mutation carriers. While limited retrospective studies evaluated the clinical value of risk-reducing contra-lateral mastectomy in early BC patients with *gBRCA1/2* mutation, showing controversial results [62,63,64], there is only one prospective study examining its benefit in TNBC patients with *gBRCA* mutation, which included a limited number of patients [18]. This study showed that contra-lateral prophylactic mastectomy conferred no survival benefit for *g*BRCA mutation carriers. *Based on the current evidence, 69% of the panel advised offering contra-lateral mastectomy as a risk reduction method and 31% opted against it. In addition, the panel highlighted the fact that such an intervention should depend on the patients’ age, stage, preference and risk of developing contra-lateral BC, rather than doctor’s recommendation solely.*

#### 4.2.4. Postoperative Radiation Therapy Indication after Mastectomy for pT1−2 N0 TNBC

Only a few studies have addressed the clinical value of adding post-mastectomy radiation therapy (PMRT) for pT1−2 N0 TNBC, and the available evidence is still controversial. Some retrospective studies showed that PMRT improved DFS compared to mastectomy alone in pT1−2 N1 cases only, but not in “N0”, while others deemed that PMRT improved outcomes also in pT1−2 N0 disease [49,65,66,67,68,69]. The largest study so far that evaluated PMRT in patients with “N0” TNBC demonstrated an OS benefit for T3 disease patients, but not for those with T1−2; however, the use of PMRT was low in the T1−2 group [65]. In a meta-analysis evaluating the value of postoperative radiation therapy in 5500 patients with TNBC, BCT and PMRT were compared to mastectomy alone. Postoperative radiation therapy was associated with a significantly lower risk of LRR, irrespective of the type of surgery, but with no OS benefit, except in late-stage disease (≥T3/≥N2) [67]. Accumulating evidence suggests that T1−2 N0 TNBC is a heterogeneous population involving some subgroups that may benefit from radiation therapy [68,70,71]. Lympho-vascular invasion (LVI) was shown to be associated with a higher risk for LRR after mastectomy in T1−T2 N0 TNBC treated without PMRT, with a 5 and 10-year LRR rate of 18.2% and >30%, respectively, and was also a predictor of worse DFS [70]. *Therefore, in an attempt to upscale the level of local control, which normally would not be offered to these early stages in other less aggressive biological BC subtypes, the panel was asked about the role of adding PMRT for pT1−2 N0 TNBC. A consensus was reached by the panel (91.6%) to reject that TNBC biology per se as an absolute indication for postoperative radiation therapy after mastectomy in these early stages. The panel pointed out that the decision to add PMRT to mastectomy in pT1−2 N0 TNBC should incorporate other risk factors for LRR, such as LVI and grade.*

#### 4.2.5. Hypo-Fractionation Radiation Therapy Regimens for TNBC

It is still unclear whether the standard fractionation and hypo-fractionation (HF) regimens have similar effects in all BC subtypes. The American Society for Radiation Oncology (ASTRO) 2018 guidelines for whole breast irradiation (WBI) recommended that the decision to offer HF may be independent of hormone and HER2 receptor status [72]. The Ontario Clinical Oncology Group (OCOG) study showed no statistically significant difference in local recurrence between HF and conventional regimen in basal tumours (HR = 1.27, 95% CI = 0.21–7.58) [73]. This study found that the histological subtype was not predictive for response to HF regimen, suggesting that it can be safe in TNBC. An obvious caveat was the limited number of TNBC patients in this study, and the lack of outcomes reporting according to molecular subtypes in START trials, which makes it challenging to conclude results. *Almost half of the panel (47%) abstained from responding to this question, which reflects the limited evidence on this matter. However, 87% of the panel agreed that HF could be used for TNBC cases, (15% voted for HF in early stages, 13% for advanced stages that need comprehensive nodal irradiation and 59% for both); 13% refused to offer HF for TNBC. While this topic requires more evidence, we cannot deny the value of HF in having good local tumour control with comparable long-term survival and safety outcomes as stated by START trials.*

#### 4.2.6. Radiation Therapy Boost for TNBC after Lumpectomy

At the time when clinical studies and guidelines were adapting boost doses to tumour bed after lumpectomy, the concept of tumour biology was still evolving, which has led to a knowledge gap in the value of boost doses according to tumour biology. As such, the benefit of tumour bed boost in TNBC specifically is yet to be well defined. However, one could speculate that adding a boost to tumour bed after WBI may provide an additional benefit in reducing the risk of local relapse [74]. The reason for this additional benefit is the fact that TNBC, which often occurs in young women with high-grade tumours, are more likely to have residual disease after lumpectomy [75], and it is well known that a radiation boost had the largest proportional benefit in patients younger than 50 years and those with high-grade tumours [76]. *Accordingly, 58% of the panellists agreed, while 42% declined to offer tumour bed boost to all cases of TNBC after BCS. This was a challenging vote with no consensus; however, voters in favour of this approach based their opinion on the observational data showing a higher incidence of LRR in TNBC tumours, which may necessitate further local treatment control measures*.

#### 4.2.7. Regional Nodal Irradiation after Upfront Surgery for pT1−3 N0 TNBC

Data are still evolving and lack robust evidence to confirm a favourable impact of postoperative regional nodal irradiation (RNI) on loco-regional control or survival in patients with pT1−3 N0 TNBC. *The panel reached a consensus of 86% that RNI in these patients should not be recommended based solely on TNBC biology. The panel discussed the fact that it is mandatory to consider other poor prognostic features including LVI and high-grade when suggesting such an indication.*

#### 4.2.8. Regional Nodal Irradiation after Mastectomy for pN1 (1–3 + LNs) TNBC

The value of RNI in BC is quite clear and is recommended by numerous guidelines for BC with four or more positive LNs [21,77]. However, indicating RNI after mastectomy for pN1 (1–3 + LNs) BC is still controversial. The ASTRO 2016 guidelines [78] did not recommend RNI for 1–3+ LNs involvement as an absolute indication. However, they specified the chest wall and supraclavicular-axillary apical nodes as a minimum mandatory target volume in current practice for these cases. The only study that reported the outcomes based on the receptor status was the MA.20 trial [79], which showed significant improvement in 10 year DFS rates in the RNI arm vs. control (76% vs. 62%, HR = 0.56, 95% CI = 0.39–0.81) and 10-year OS rates (81% vs. 74%, HR = 0.69, 95% CI= 0.47–1) respectively, in the ER− tumours. However, the benefit of this treatment option was not addressed for TNBC specifically but was significant for ER− tumours, which represented 25% of the whole cohort (230 patients). *Based on the proposed higher LRR with TNBC biology and the needed evidence in this area, 76% of the panellists preferred including regional lymph nodes in radiation therapy fields for TNBC with 1–3 LNs involvement.*

#### 4.2.9. Axillary LN Dissection Versus Observation in TNBC Patients with Z-0011 Criteria (cT1−2N0, BCS and 1–2 + SLNB)

The Z-0011 study investigated the benefit of axillary LN dissection (ALND) vs. observation in patients with cT1–2 tumours and positive 1−2 sentinel lymph nodes (SLN), demonstrating that 10 year OS for the observational arm was non-inferior to the ALND arm [80]. However, this study had some limitations, including the lack of reporting of the HER2 status and the exclusion of patients who received NAT. *Based on this evidence, 31% of the panellists voted to keep patients with TNBC with the Z-0011 criteria under observation, while 46% of the panellists voted for ALND in these patients, keeping with the observational data of higher regional recurrences seen with TNBC tumours, along with the under-presentation (15%) of ER/PR− population in the Z-0011 study. Nevertheless, 23% voted for axillary radiation for these patients, stating that the tangential radiation fields used in this study delivered a considerable dose to the axilla, but not as high as delivering a full axillary planned dose.*

#### 4.2.10. Reconstruction Timing after Breast Surgery for TNBC

A study by Morrow et al. assessed the outcomes for patients who underwent delayed vs. immediate breast reconstruction for BC [81]. They reported that both approaches had comparable survival and local recurrence rates. Another prospective study looked at 1806 patients who underwent immediate vs. 151 patients who underwent delayed reconstruction and reported that the delayed approach had less incidence of complications (Odds Ratio (OR) = 0.38, *p* < 0.001); however, patients and surgeons were still in favour of immediate reconstruction [82]. *Although compelling data are pointing to higher LRR rates in TNBC* vs. *non-TNBC, in the absence of clinical evidence, cancer recurrence seems to develop independently of the type of the reconstruction procedure. Based on the available evidence, 68% of the panellists had no specific preference on either approach, while 18% and 14% voted for immediate and delayed reconstruction, respectively, as accepted approaches.*

### 4.3. Neo-Adjuvant Therapy (NAT) for TNBC

#### 4.3.1. Neo-Adjuvant Therapy for Early-Stage TNBC (cT2–3 N0–1)

While the use of NAT for locally advanced stages is the standard of care, it is increasingly being considered for early BC [83], with its impact on long-term outcomes being controversial. The main aims of NAT are to reduce the extent of surgery in the breast and/or axilla, to attain the good prognostic impact of pCR and to guide the adjuvant therapy according to response. Besides allowing for more breast tissue conservative surgery, NAT may offer to omit the formal axillary dissection in patients with limited axillary tumour burden (cN1) who showed a clinical complete response from applying strict technical standards [83]. Additionally, data showed that achieving a pCR is associated with better DFS and OS in TNBC [84,85,86]. Unfortunately, this occurs in approximately one-third of patients with TNBC treated with neo-adjuvant chemotherapy (pCR range 17–53%), while the majority with non-pCR are correlated to poorer outcomes [86,87]. Recently the CREATE-X trial showed a survival benefit for using capecitabine as adjuvant therapy for TNBC patients who do not achieve pCR. This, in turn, would add more value to use NAT, as it may guide tailoring the adjuvant therapy based on response [88]. *The majority of the panellists (93%) supported the use of NAT for cT2–3 N0 TNBC, regardless of the planned surgery type. This agreement to offer NAT was based on the ability to downstage the tumor, the prognostic value of pCR, the ability to de-escalate the surgical approach and adjuvant therapy, and the window it provides for research. On the other hand, in the case of early TNBC cases with N1 disease (cT2–3, N1), all the panellists (100%) outweighed the value of NAT as well.*

#### 4.3.2. Number of Cycles in The Neo-Adjuvant Setting for TNBC

The ideal number of cycles in the neo-adjuvant setting is still controversial. In real-life clinical practice, it may be argued that there should not be a limit to the number of cycles but rather to be guided by the maximum response of the tumour. *Imaging is advised midway through the NAT to assess response and to exclude progressive disease [83]. Seventy-six percent of the panellists voted for 6–8 cycles, which is still aligned with most of the reported study designs, whereas 24% of the voters preferred to choose a more ’dynamic’ approach where the number of cycles would be guided by the toxicity and maximum tumour response achieved before referral to surgery.*

#### 4.3.3. Neo-Adjuvant Regimen of Choice for TNBC

In the seminal meta-analysis (CTNeoBC), an anthracycline and taxane-based NAT were associated with pCR in 34% of TNBC patients, with a significant association between the achievement of pCR and event-free survival (EFS) [84]. Since then, anthracycline and taxane-based therapy have been considered the mainstay in the neo-adjuvant setting for TNBC in all subsequent trials [89,90]. To improve the pCR further, the “phase II” CALGB 40603 trial reported significantly higher pCR rates with carboplatin plus paclitaxel in TNBC patients, at a pCR rate of 54% vs. 41% with paclitaxel alone [91]. Similar results were reported by the “phase II” GeparSixto [92], and “phase III” BrighTNess studies [93], where pCR rates were 53% vs. 37% and 58% vs. 31% with platinum-containing vs. non-platinum NAT regimens, respectively. However, it is important to note that none of these studies reported a significant OS benefit, partly due to the small sample size. Nevertheless, the GeparSixto trial exceptionally showed a statistically significant improvement in 3-year DFS with the addition of carboplatin (86% vs. 76%, HR = 0.5). A recent meta-analysis confirmed that platinum-based therapy was associated with significant improvement in pCR vs. non-platinum-based therapy (40% vs. 27%, OR = 1.75, 95% CI = 1.36–2.62) [94]. As expected, the beneficial effect of carboplatin was associated with increased haematological toxicity [91,92]. *Based on the previous data, 77.6% of the panellists voted for anthracycline/taxane/platinum as the preferred regimen in the neo-adjuvant setting for TNBC, and 22.4% voted for anthracycline /taxane only. However, the panel stated that the final verdict regarding adding platinum in this context still needs well-designed studies, with long term follow-up. Moreover, they proposed that adding platinum to a taxane in the NAT is especially preferred in patients with stage II–III TNBC, and if suboptimal tumour response was achieved following anthracyclines. However, 86% of the panellists declined de-escalating the neo-adjuvant chemotherapy in TNBC by offering a short, anthracycline-free, taxane/platinum regimen only, for early responders (response-adapted approach, ADAPT-TN trial [95]), while 14% may consider this approach in low-risk patients.*

#### 4.3.4. Platinum-Containing NAT for TNBC According to *BRCA* Status

Only few studies analysed the impact of including platinum-based NAT on clinical outcomes according to *BRCA* status in TNBC [96]. Among the *gBRCA* mutation carriers in the CALGB 40603 study, the pCR rate was not further impacted by the addition of carboplatin [91,97]. In the GeparSixto trial, carboplatin significantly increased pCR and DFS for the whole cohort of TNBC; however, in the *gBRCA* mutant subgroup, adding carboplatin did not improve pCR nor DFS [92]. In the BrighTNess trial, which included 634 TNBC patients (15% had *gBRCA* mutations) the addition of carboplatin to standard NAT (12 weekly paclitaxel followed by four cycles doxorubicin and cyclophosphamide (AC)) significantly improved pCR compared to standard chemotherapy alone (58% vs. 31%, respectively). Importantly, the magnitude of pCR benefit with the addition of carboplatin was more seen in patients with *BRCA* wild type (*wBRCA)* (59% vs. 29%), rather than those with *gBRCA* mutation (50% vs. 41%). A third arm in this study included patients who received Veliparib (a poly-adenosine diphosphate-ribose polymerase inhibitor (PARP inhibitor)) in addition to carboplatin and paclitaxel, did not significantly improve the pCR rate over the combination of carboplatin and paclitaxel in patients with *gBRCA* mutation (57% vs. 50%, respectively) [93].

Whether the addition of neo-adjuvant platinum is preferentially more beneficial in patients with *gBRCA* mutation compared to those with *wBRCA* was addressed by a recent meta-analysis, suggesting that the addition of platinum did not significantly improve the pCR rate in TNBC patients with *gBRCA* mutation vs. those with *wBRCA* (*p* = 0.08) [96]. *Due to the lack of high-quality evidence, the panel did not reach a conclusion regarding adding platinum in the NAT based on BRCA status. In TNBC with gBRCA mutation, 69.2% of the panellists preferred platinum incorporation in NAT, while in wBRCA TNBC, 66.6% opted against its use. The final answer of the exact role of neo-adjuvant platinum in patients with TNBC will probably be provided by the PEARLY trial, which is a large (n = 840 patients) ongoing phase III trial aiming to compare four AC followed by four taxanes with or without carboplatin (AUC 5). Patients in this study are stratified according to BRCA status. The primary objective is 5-year event-free survival, and data are expected to be presented in 2023 [98].*

#### 4.3.5. Immunotherapy in The Neo-Adjuvant Setting in Early TNBC

The recent phase III, Keynote 552 study included 1200 patients with early-stage TNBC who were randomized to receive chemotherapy (12 weekly paclitaxel/carboplatin followed by four cycles anthracycline/cyclophosphamide) +/− Pembrolizumab in the neo-adjuvant phase. After definitive surgery, patients received Pembrolizumab or placebo for nine cycles or until recurrence or unacceptable toxicity (adjuvant phase). After a median follow-up of 15.5 months, the addition of Pembrolizumab in NAT significantly increased the pCR rate (64.8% vs. 51.2%) with a trend for favourable EFS with just 9% of the events recorded [99]. It should be noted that the KEYNOTE 522 is the only neo-adjuvant trial for TNBC powered for EFS. IMpassion031 [100] is a smaller randomized phase III trial that included 333 patients with early-stage TNBC. Patients were randomized to receive chemotherapy (12 weekly nab-paclitaxel followed by four cycles AC) +/− Atezolizumab. Atezolizumab was continued after surgery to complete one year of therapy. Atezolizumab was associated with a significant increase in pCR (57.6% vs. 41.1%, *p* = 0.004). For both studies, the improvement in pCR was seen regardless of PD-L1 status and was greater in patients with node-positive disease, though immune-related toxicities remain of significant concern in those patients with potentially curable disease and longer-term survival results should be awaited. On the other hand, Atezolizumab, when added to neo-adjuvant chemotherapy (nab-paclitaxel/carboplatin), failed to increase pCR in the NeoTRIP trial [101]. *The panel did not reach a consensus regarding the incorporation of Pembrolizumab or Atezolizumab in the neo-adjuvant setting. Seventy-two percent of the panellists stated that these agents should not be used off-label in the neo-adjuvang setting, as there are no mature data yet. However, 28% of the panellists favored its use in high-risk early-TNBC patients.*

### 4.4. Adjuvant Therapy for TNBC

#### 4.4.1. Adjuvant Treatment in the Absence/Presence of pCR after NAT (Anthracycline/Taxane) in TNBC

Patients with TNBC with residual disease following neo-adjuvant anthracycline/taxane have a relapse rate of more than 40% at 5 years [84]. The open-labelled, randomized controlled trial “CREATE-X” evaluated the role of adjuvant capecitabine among Her2− patients who had residual invasive carcinoma after NAT. The DFS was significantly higher in the capecitabine vs. control group (69.8% vs. 56%, respectively, HR = 0.58). Of note, the benefit was most evident in the TNBC subgroup (30% of the cohort), where the 5-year DFS was 70% vs. 56% with capecitabine vs. control, respectively. In addition, the OS was significantly improved (78.8% vs. 70.3%, respectively, HR = 0.52, 95% CI = 0.30–0.90) [88]. The caveat of this study was that the data for TNBC were from a subgroup analysis. Still, whether adjuvant capecitabine in this context is better than adjuvant platinum-based chemotherapy remains an intriguing question. The EA 1131 (NTC02445391) [102] is an ongoing randomized phase III trial comparing four cycles of platinum (cisplatin or carboplatin) vs. six cycles of capecitabine as adjuvant therapy in TNBC patients with postoperative residual disease (≥10 mm invasive disease) following NAT. Residual disease should be of basal-like subtype as determined by PAM50 assay prior to randomization. The estimated study completion date is May 2024. *A consensus was reached by the panel (85%) recommending adjuvant capecitabine for 6–8 cycles in case of absence of pCR following neo-adjuvant anthracycline and taxane regimen. On the other hand, in the case of the presence of pCR after NAT, a consensus agreement (93%) was reached accepting that no further adjuvant therapy is needed.*

#### 4.4.2. Adjuvant Chemotherapy after Upfront Surgery in pT1a–b N0 TNBC

A prospective study included 363 patients with T1a–b N0 TNBC had illustrated the favourable natural history of these small tumours. It showed that without chemotherapy, the 5-year breast cancer-specific survival (BCSS), DFS, and OS were 95%, 93% and 94%, respectively, for T1a N0 tumours, and 95%, 90% and 91% for T1b N0 tumours, respectively [103]. This was confirmed by a recent SEER database analysis of more than 9000 stage I, TNBC patients. Patients with T1a–b N0 who received chemotherapy did not show significantly better BCSS or OS than those who did not receive it. While patients who received chemotherapy with poorly differentiated tumours had better BCSS (HR =  0.68, 95% CI = 0.52–0.88) and OS (HR =  0.54, 95% CI= 0.44–0.66) compared to patients who did not receive it [104]. Notably, a recent meta-analysis (2020), demonstrated a significant benefit of adjuvant chemotherapy among patients with T1b disease (RR = 0.62, 95% CI= 0.42–0.92), but not for patients with T1a disease (RR = 0.64, 95% CI = 0.31–1.33) [105]. *Given the good prognosis, the majority (88%) of the panel accepted omitting adjuvant therapy for pT1a N0 TNBC, while 12% preferred offering adjuvant chemotherapy in these cases. On the other hand, 97% of the panel opted for adjuvant chemotherapy for pT1b N0 TNBC.*

#### 4.4.3. Adjuvant Regimen after Upfront Surgery for Stages I–III TNBC

The benefit of adding taxanes to anthracyclines in the adjuvant treatment of BC is well-established [106,107]. In patients with node-positive disease, the addition of taxanes in the CALGB 9344/INT1048 study [106] showed a 17% reduction in the risk of recurrence, with an improvement in 5-year DFS and OS from 65% to 70% and 77% to 80%, respectively. On the other hand, incorporating platinum agents in the adjuvant treatment of TNBC is controversial. Interestingly, a recent phase III “Pattern study” [108] had randomized 647 patients with early TNBC (74% were node-negative) to either paclitaxel-carboplatin or cyclophosphamide, epirubicin and fluorouracil followed by docetaxel, postoperatively. The 5-year DFS was statistically significantly higher in the carboplatin group (86.5% vs. 80.3%; HR =  0.65; 95% CI = 0.44–0.96; *p* =  0.03); however, no statistical difference in OS. *The panel reached a consensus (80%) regarding the preferred adjuvant chemotherapy regimen for stage I TNBC after upfront surgery being anthracyclines/taxanes for six cycles. Regarding adjuvant treatment for stage II–III TNBC after upfront surgery, 61% of the panel recommended 6–8 cycles of anthracyclines/taxanes and 39%* *favored the benefit of adding platinum to this regimen.*

#### 4.4.4. Dose-Dense Doxorubicin and Cyclophosphamide Followed by Paclitaxel (AC-T) in The Adjuvant Setting of TNBC

**Figure 1:**
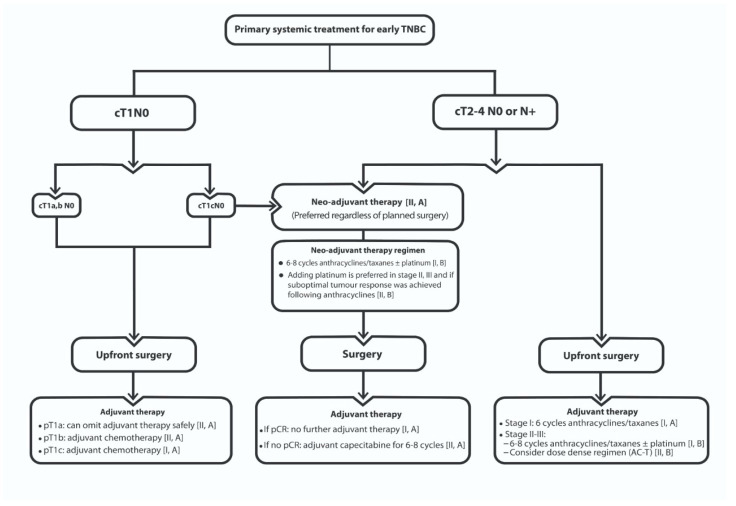
Primary systemic therapy algorithm for early TNBC.

The Early Breast Cancer Trialists’ Collaborative Group (EBCTCG) meta-analysis compared bi-weekly vs. standard 3-weekly schedules, including 15,512 BC patients with a median follow-up of 7.4 years [109]. Dose-dense regimens were associated with a significant 4.4% absolute reduction in recurrence (*p* < 0.0001) and 2.9% reduction in 10-year BC mortality (*p* = 0.0004). Notably, the proportional recurrence reductions were similar for patients with positive 1–3 LNs, ≥4 LNs and appeared to be similar in node-negative disease also. The routine use of prophylactic G-CSF, allowed delivery of dose-dense regimens without any significant increase in short-term and long-term morbidity or mortality. *Based on these data, the majority (88%) of the panel endorsed the beneficial effect of dose-dense regimens in TNBC. However, in the light of lack of prospective studies focusing on the long-term benefit of this regimen in TNBC, the panel was split on its individualized indication in the adjuvant setting for TNBC. Fifty-one percent of the panel advocated the dose-dense AC-T for patients at high risk of recurrence, while 37% expanded its indication for all TNBC cases who will receive chemotherapy, and 12% did not recommend it owing to its expected toxicity. Figure 1 depicts the treatment algorithm proposed for primary systemic therapy for early TNBC.*

### 4.5. The Metastatic Setting for TNBC

#### 4.5.1. Tissue Biopsy and Biomarkers Evaluation in TNBC Cases Developing Metastasis

Retesting for ER, PR and HER2 are recommended to account for possible discordance between the primary and secondary tumours that may reach 13% for ER− to ER+, 16% for PR− to PR+ and 4.6% for HER2− to HER2+, as shown by the pooled analysis of two prospective studies [110]. Testing for PD-L1 and *gBRCA* mutations is considered in order to select patient candidates for immunotherapy and PARP inhibitors, respectively, if these agents are available. *The panel reached a consensus (95%) supporting tissue biopsy if TNBC case had developed metastasis to evaluate these tissue biomarkers that may guide further therapy.*

#### 4.5.2. Chemotherapy after Complete Resection of Isolated Loco-Regional Recurrence in Non-Metastatic TNBC

The CALOR trial [111] enrolled 162 patients (58 patients with ER− isolated LRR) to be randomized to receive chemotherapy or not, after complete resection. In the ER− subgroup, DFS was substantially improved in the chemotherapy arm (10-year DFS = 70% vs. 34% with chemotherapy vs. not, respectively; HR = 0.29, 95% CI = 0.13–0.67). The choice of chemotherapy was left to the discretion of treating physicians, based on prior exposure and toxicity, where the majority of patients received poly-chemotherapy. *After complete resection of isolated LRR in non-metastatic TNBC, the majority of the panel (85%) recommended “pseudo-adjuvant” chemotherapy for 3–6 months.*

#### 4.5.3. Categorization of Metastatic TNBC (mTNBC)

Although chemotherapy has been the main treatment station for mTNBC for a long time, this practice has changed recently with the advent of immunotherapy for patients with PD-L1+ tumours, and with the results of PARP inhibitors for patients with *gBRCA* mutation. *The majority (89%) of the panellists agreed that mTNBC is a heterogeneous disease and should be categorized as: (1) PD-L1+ mTNBC; (2) gBRCA mutant mTNBC; (3) PD-L1−/wBRCA mTNBC or no testing done.*

#### 4.5.4. First-Line Therapy for PD-L1+, Unresectable, Locally Advanced/mTNBC

In 2019, the FDA granted accelerated approval to Atezolizumab in combination with nab-paclitaxel for patients with unresectable locally advanced/mTNBC, expressing PD-L1 ≥1%, as determined by Ventana SP142 IHC assay [112]. This was based on the IMpassion130 study [113] showing significant efficacy results driven by the PD-L1+ population in patients with recurrence of disease at least 12 months from their last therapy in the early-stage setting. This study was a phase III study, randomized 902 patients with untreated locally advanced/mTNBC to nab-paclitaxel with Atezolizumab or placebo. After median follow up of 12.9 months, in the intention-to-treat analysis (ITT), the median progression-free survival (PFS) was 7.2 months with Atezolizumab vs. 5.5 months with placebo (HR = 0.80, 95% CI= 0.69–0.92, *p* = 0.002). Among patients with PD-L1+ tumours (41% of the ITT population), the median PFS was 7.5 months and 5.0 months, respectively (HR = 0.62, *p* < 0.001). In the 2nd interim OS analysis [114], no statistical significance was demonstrated in ITT patients. Importantly, treatment-related grade 3 or higher adverse effects were higher in Atezolizumab vs. placebo arm (40% vs. 30%). The final survival analysis presented at ESMO 2020 demonstrated a clinically meaningful absolute survival improvement of 7.5 months in the PD-L1+ population, translating into 3-year OS rates of 36% vs. 22% (HR = 0.67, 95% CI = 0.53–0.86), which was not tested statistically due to the hierarchical statistical design of the study [115].

The IMpassion131 trial was presented also at the ESMO 2020 [116]. In this study, patients with mTNBC were randomized to receive paclitaxel with or without Atezolizumab as first-line therapy. In this study, there was no benefit from the addition of Atezolizumab even in the PD-L1+ population. The difference in results of the two Atezolizumab studies is puzzling and should not be ignored. The only explanation to date is the difference in chemotherapeutic partner, although the results from KEYNOTE 355 [117] suggest otherwise.

KEYNOTE 355 results were presented in ASCO 2020, leading to the accelerated approval of Pembrolizumab in combination with chemotherapy (paclitaxel, nab-paclitaxel, or gemcitabine/carboplatin) for previously untreated patients with locally recurrent unresectable or mTNBC whose tumours express PD-L1 (combined positive score [CPS] ≥ 10) as determined by PD-L1 IHC 22C3 pharmDx test [117]. In this study, 847 patients were randomized to chemotherapy with or without Pembrolizumab. After a median follow up of 26 months, patients with CPS ≥ 10 (38% of the ITT population) had a median PFS of 9.7 months with Pembrolizumab vs. 5.6 months with placebo (HR = 0.65, 95% CI = 0.49–0.86, *p* = 0·0012). However, median PFS was not significantly improved among patients with CPS≥ 1 or ITT population. Grade 3–5 treatment-related adverse event rates were 68% with Pembrolizumab vs. 67% with placebo. A key strength in this study is that in addition to patients who had de novo metastatic disease, this study included patients with early recurrences (relapsed at ≥ 6 months from their early-stage therapy), which is a rather common scenario in TNBC. Moreover, it highlighted the ability of Pembrolizumab to work with paclitaxel as well as nab-paclitaxel.

*Based on these data, the majority (86%) of the panellists voted for Atezolizumab + nab-paclitaxel or Pembrolizumab + chemotherapy as a preferred option over standard chemotherapy in the first-line setting for unresectable locally advanced/mTNBC expressing PD-L1 (PDL-1 ≥ 1% for Atezolizumab or CPS ≥ 10 for Pembrolizumab), while only 14% felt that it could be used in any line of therapy, hopefully extrapolating the beneficial clinical effect to subsequent lines, in spite of the lack of supporting clinical evidence. Moreover, for the first-line immunotherapy, 58% of the panellists did not prefer one over the other (Atezolizumab/Pembrolizumab), as no head-to-head comparison, while 22% preferred Atezolizumab + nab-paclitaxel, owing to the absolute survival improvement of 7.5 months in the PD-L1+ population (≥ 1%) and 20% favored Pembrolizumab in PD-L1+ (CPS ≥10), weighing the longer follow up study and that it can be combined with many chemotherapeutic partners.*

#### 4.5.5. Immunotherapy in Subsequent Lines for PD-L1+, mTNBC

In the open-labelled phase III study “Keynote-119”, 622 previously treated mTNBC patients were randomized to Pembrolizumab monotherapy vs. single-agent chemotherapy. After 9.9 months, Pembrolizumab did not significantly improve PFS nor OS in patients with CPS ≥ 10 or CPS ≥ 1 or in the whole arm. However, in the exploratory analysis, Pembrolizumab therapy effect increased as CPS increased, with median OS being 14.9 months vs. 12.5 months with Pembrolizumab vs. chemotherapy, respectively (HR = 0.58, 95% CI = 0.38–0.88), in patients with CPS ≥ 20 [118]. *The panel uniformly agreed (93%) after discussion that the role of Pembrolizumab in the subsequent lines of therapy in the metastatic setting is yet to be defined and needs more evidence to be validated*

#### 4.5.6. PD-L1 Testing in Unresectable Locally Advanced/mTNBC

In the IMpassion130 trial, representative tumour specimens (paraffin-embedded archival or fresh pre-treatment relapsed-disease tumour tissue) were evaluated by central testing of PD-L1 expression using SP142 IHC assay, Ventana Medical Systems. Patients were stratified based on PD-L1 expression on tumour-infiltrating immune cells as a percentage of tumour area (<1% [PD-L1−] vs. ≥1% [PD-L1+]) according to the scoring system described by Vennapusa et al. [119]. The concordance of SP142 with two other commonly used PD-L1 IHC assays and their ability to predict clinical activity of Atezolizumab were analyzed in an exploratory post hoc analysis. Available samples from IMpassion130 (614 patients in the ITT population, 68%) were evaluated for PD-L1 status using SP142 or SP263 IHC assays (SP142 or SP263 positive = ≥ 1%) or 22C3 assay (22C3 positive = CPS ≥ 1) in a central laboratory. The prevalence of PD-L1+ was 46%, 75% and 81% with SP142, SP263 and 22C3 assays, respectively. The overall percentage agreement of SP142 with SP263 and 22C3 was 69% and 64%, respectively. Intriguingly, the benefit in PFS and OS from the addition of Atezolizumab in SP142−/SP263+ or SP142−/22C3+ subgroups was smaller than in SP142+ group [120]. These data reinforced the use of the SP142 assay for PD-L1 testing to define PD-L1−positive status adopted in the IMpassion130 trial when selecting patients with advanced TNBC who will obtain the greatest benefit from adding Atezolizumab to standard first-line chemotherapy. *Based on these data, the panel agreed with a consensus (93%) that Atezolizumab plus nab-paclitaxel should be offered for unresectable locally advanced/mTNBC patients after PD-L1 testing to identify the PD-L1+ population. Furthermore, if Atezolizumab is planned, the panel (94%) confirmed that PD-L1 should be tested using the Ventana SP142 IHC on the tumoural immune cells and that the cut-off value for PD-L1 positivity is 1%. On the contrary, if Pembrolizumab is planned, the panel (93%) acknowledged PD-L1 testing by 22C3 pharmDx test as demonstrated in the Keynote 355, with the cutoff of CPS ≥ 10 [117].*

#### 4.5.7. Germline Mutant *BRCA1/2* mTNBC

Platinum chemotherapies (carboplatin/cisplatin) were considered the preferred agents for treating mTNBC with *gBRCA*1/2 mutation based on the concept of inability to repair the platinum induced DNA damage [121,122]. In the phase III “TNT trial” [123], 376 patients with mTNBC were randomized to receive carboplatin or docetaxel in the first-line setting. There was no statistically significant difference in the overall response rate (ORR) with carboplatin or docetaxel (31.4% vs. 34%, respectively; *p* = 0.66) or survival improvement in the overall population. However, patients with *gBRCA* mutation (n = 43) showed significantly better response to carboplatin than to docetaxel (ORR = 68% vs. 33.3%, *p* = 0.03). Moreover, PFS was improved with carboplatin (6.8 vs. 4.4 months; *p* = 0.002), although no difference in OS was observed with any of the agents. Two recent studies evaluated the benefit of PARP inhibitors in *gBRCA* mutant advanced BC showing efficacy with tolerable side effects, which led to their FDA approval in 2018. The OlympiAD study [124] enrolled 302 patients with *gBRCA* mutant, HER2− metastatic BC who had received ≤2 metastatic lines of chemotherapy. Patients were randomized to Olaparib or treatment of physician’s choice (TPC). Median PFS was significantly longer in the Olaparib group vs. TPC (7.0 vs. 4.2 months, HR = 0.58, *p* < 0.001). After a median follow up of 25.8 months, there was no observed difference in OS, and the HR for OS was 0.5 in the pre-specified subgroup with no prior chemotherapy for metastatic BC. The EMBRACA trial [125,126] was a phase III trial that enrolled 431 participants with *gBRCA* mutant, HER2− advanced BC who received ≤3 metastatic lines of chemotherapy. Patients were randomized to Talazoparib or TPC, with significant improvement in median PFS with Talazoparib compared to TPC (8.6 vs. 5.6 months). Specifically among TNBC patients, PFS was significantly higher with Talazoparib than TPC (HR = 0.60, *p* = 0.007). Soon after the consensus meeting was held, the OS update of the EMBRACA was presented at ASCO 2020, showing that Talazoparib did not improve OS significantly [127]. It should be noted that the ORR was doubled with PARP inhibitors (60% vs. 29% with Olaparib vs. TPC and 62% vs. 27% with Talazoparib vs. TPC, respectively). Moreover, the median time to response was comparable with PARP inhibitors to chemotherapy, being similar in both arms of OlympiAD trial (1.5 months), and 2.6 vs. 1.7 months with Talazoparib vs. TPC, respectively, which highlights PARP inhibitors as an appealing alternative to chemotherapy when rapid response is needed in patients with a high burden of disease. Worth mentioning that PARP inhibitors were introduced as a 1st line therapy in 29% and 39% of the patients in the OlympiAD and EMBRACA studies, respectively.

*Based on the mentioned data, there was a split of voting due to the lack of data regarding the most efficacious sequencing of systemic therapy agents in gBRCA mutant mTNBC or trials comparing PARP inhibitors vs. platinum in the first or subsequent lines of therapy in this group of patients; taking into account the comparable ORR and PFS results recorded by both the carboplatin in TNT trial and PARP inhibitors in OlympiAD and EMBRACA studies. For gBRCA1/2 mutant mTNBC, 66% of the panel preferred a platinum or PARP inhibitor to be incorporated early in the treatment, 22% voted that the most efficacious sequencing has yet to be defined and 12% supported anthracyclines and taxanes to be the mainstay of therapy. For carboplatin, 42% of the panellists preferred its use in the first line for gBRCA mutant mTNBC, while 58% recommended it in subsequent lines of therapy only. For Olaparib, 69% of the panellists voted that it could be the preferred option in any line of therapy, while 31% recommended it in subsequent lines of therapy only. For Talazoparib, 72% of the panel also stated that it could be a preferred option in any line of therapy, while 28% recommended it in subsequent lines of therapy only.*

In the setting of combining PARP inhibitors with platinum, the BROCADE 3 trial randomized 509 patients with *gBRCA* mutant HER2− advanced BC who received ≤2 metastatic lines of chemotherapy to receive carboplatin and paclitaxel with or without Veliparib. Median PFS was 14.1 months vs. 12.3 months, respectively (HR = 0.71, *p* = 0.002) [128]. *To date, Veliparib is still not FDA-approved, which supported the decision of a vast majority (86%) of the panellists not to consider Veliparib as an option for subsequent lines of therapy for gBRCA mutant mTNBC until the presence of mature data to support its use*.

#### 4.5.8. PD-L1−/*wBRCA* or No Testing Done for mTNBC

**Figure 2:**
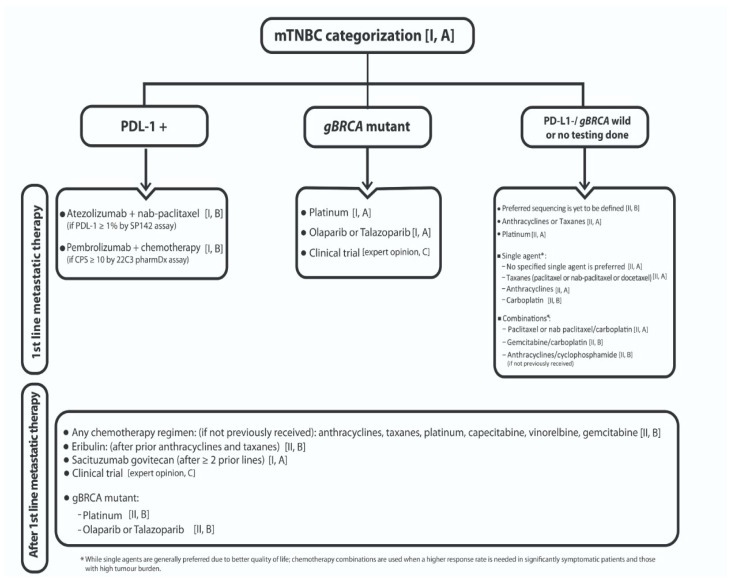
The treatment algorithm for metastatic TNBC according to subtype.

The roles of anthracyclines and taxanes in the first-line metastatic setting for treatment of mTNBC are well known [129], either as single or combination regimens. While single agents are generally preferred due to better quality of life, chemotherapy combinations are used when a higher response rate is needed for significantly symptomatic patients and those with high tumour burden as recommended by international guidelines [130]. However, the value of platinum in non-mutant *BRCA* mTNBC patients needs validation [122]. In the TNT study, as previously mentioned, carboplatin was better tolerated but not more active than docetaxel in terms of ORR, PFS or OS in the whole population. Remember that the subgroup of *gBRCA* mutant patients who gained benefit by the addition of platinum represented only 11% (43/376) of the whole population [123]. *Regarding the most efficacious sequencing of chemotherapy agents for mTNBC with PDL1−/wBRCA or if no testing was done, 50% of the panel stated that it has yet to be defined, 30% recommended early incorporation of anthracyclines/taxanes in the patient’s treatment course and 20% supported using platinum as the first-line regimen. In case that a single-agent chemotherapy will be used in the first line, 35% preferred paclitaxel or nab-paclitaxel or docetaxel, 10% voted for carboplatin, 10% for anthracyclines and 45% defined that no specified single agent is preferred in the first-line. Nevertheless, if a combination chemotherapy will be used in the first line, 60% voted for paclitaxel or nab-paclitaxel/carboplatin as the preferred regimen, while 20% recommended gemcitabine/carboplatin and 20% encouraged starting with anthracycline/cyclophosphamide in first-line, if not previously received. For subsequent lines, no preferred chemotherapy regimen was identified and enrolment in clinical trials is encouraged (75%). Figure 2 represents the treatment algorithm proposed for metastatic TNBC.*

#### 4.5.9. Eribulin for mTNBC with PD-L1−/*wBRCA* or No Testing Done

In a pooled analysis of two phase III studies evaluating eribulin in patients previously treated with anthracyclines and taxanes [131], 1864 patients were randomized to eribulin or chemotherapy. In TNBC patients (n = 428, 23%), median OS was 12.9 vs. 8.2 months with eribulin vs. chemotherapy, respectively (HR = 0.74, 95% CI = 0.60–0.92). *In patients with PDL1−/wBRCA mTNBC or who were not tested, 75% of the panel experts believed that eribulin is a preferred subsequent line of therapy after prior anthracyclines and taxanes.*

#### 4.5.10. AR-Directed Therapy in AR+ mTNBC

As approximately 10–40% of TNBC tumours express AR, it seems like an attractive target for therapy [25,26]. In mTNBC, the anti-tumour activity of AR-directed therapy is still under evaluation, with preliminary data [22,23,24] showing modest clinical benefit. *The majority of the panel (79%) agreed that AR-directed therapies are not recommended for the management of mTNBC outside clinical trials.*

#### 4.5.11. The Drug Conjugate “Sacituzumab Govitecan” for Pretreated mTNBC

Sacituzumab govitecan is a novel antibody–drug conjugate that comprises an anti-Trop-2 antibody coupled to SN-38, the active metabolite of irinotecan, enabling the intracellular release of SN-38 along with bystander effects within the tumour microenvironment. The accelerated approval by the FDA for the use of sacituzumab in relapsed or refractory mTNBC in April 2020 was based on the results of a phase 2 study, then the results of the open-label, phase III, “ASCENT trial” [132] was presented. The study compared sacituzumab with single-agent TPC (capecitabine, eribulin, vinorelbine or gemcitabine) in 468 patients with relapsed/refractory mTNBC after ≥2 prior chemotherapies for metastatic disease. Median PFS and OS were significantly longer with sacituzumab (5.6 vs. 1.7 months, HR = 0.4, *p* < 0.0001) and (12.1 vs. 6.7 months, HR = 0.48, *p* < 0.0001), respectively, with a manageable safety profile. *Accordingly, a consensus was reached by the panel (86%), preferring sacituzumab over chemotherapy after ≥2 prior chemotherapies for metastatic disease.*

## 5. Conclusions

The BGICC consensus has covered many important aspects of TNBC management, starting from defining TNBC tumours to the management of metastatic TNBC. Table 4 illustrates the levels of evidence and grades of recommendation of the consensus statements. A consensus was reached regarding the role of NAT in early TNBC with the incorporation of platinum suggested in some settings; the loco-regional management, including the use of hypo-fractionated regimens; tailoring adjuvant therapy according to the residual disease after NAT; and the role of platinum-based chemotherapy and immunotherapy in the management of advanced TNBC. Many areas for future research were identified including the role of androgen-receptor-targeted therapy, the predictive value of tumour-infiltrating lymphocytes and Ki-67 and their role in tailoring treatment, the role of platinum-based chemotherapy in the neo-adjuvant setting and the role of anti PD-L1 treatment in the management of early and advanced TNBC.

## Figures and Tables

**Table 1:** List of panellists (Alphabetical).

1. Aapro M. 2. Abdel Azim H. 3. Abdel Aziz H. 4. Abdel Karim K. 5. Abulkhair O. 6. Al-Sukhun S. 7. Anderson B.O. 8. Arun B. 9. Balch C.M. 10. Conte P. 11. ElGhazaly H. 12. ElMahdy M.	13. El Saghir N.S. 14. El-Zawahry H.M. 15. Foheidi M. 16. Frolova M. 17. Ghosn M. 18. Giuliano A.E. 19. Gligorov J. 20. Guarneri V. 21. Gulluoglu B.M. 22. Kandil A. 23. Leung J.W.T. 24. Orecchia R.	25. Paltuev R.M. 26. Penault-Llorca F. 27. Perez E.A. 28. Poortmans P. 29. Rugo H.S. 30. Sabry M. 31. Shehata M.A. 32. Shenawi M. 33. Swain S.M. 34. Yang W. 35. Yip C.H.

**Table 2:** Grades of Recommendation.

Grade	Recommendation	Grades of Recommendation
A	Strongly recommended	Strong evidence for efficacy with a substantial clinical benefit.
B	Generally recommended	Strong or moderate evidence for efficacy but with a limited clinical benefit.
C	Optional	Insufficient evidence for efficacy or benefit does not outweigh the risk or the disadvantages (adverse events, costs, etc.).
D	Generally, not recommended	Moderate evidence against efficacy or for adverse outcome.
E	Never recommended	Strong evidence against efficacy or for adverse outcome.

**Table 3:** Levels of evidence.

Level	Levels of Evidence
I	Evidence from at least one large randomized, controlled trial of good methodological quality (low potential for bias) or meta-analyses of well-conducted randomized trials without heterogeneity.
II	Small, randomized trials or large randomized trials with suspicion of bias (lower methodological quality) or meta-analyses of such trials or of trials with demonstrated heterogeneity.
III	Prospective cohort studies.
IV	Retrospective cohort studies or case–control studies.
V	Studies without a control group, case reports, expert opinions.

**Table 4:** Levels of evidence and grades of recommendation of the consensus meeting statements.

Statements	Percentage of Consensus Votes	Level of Evidence (LOE)	Grade of Recommendation (GOR)
TNBC disease is defined as HER2− with ER and PR expression <1%.	**85.5%**	I	A
HER2− and ER/PR expression between 1–10% tumours would be treated clinically as TNBC, being not eligible to receive endocrine therapy as a monotherapy.	61.5%	IV	C
Germline *BRCA* mutation testing for TNBC patients is indicated if diagnosed at ≤60 years or with strong family history in order to plan for genetic counselling and risk reduction measures for the patient and her family.	67%	II	B
Androgen receptor (AR) reporting has no current role in TNBC management plan and should be reported in TNBC cases for research purposes only.	**77%**	II	B
TILs reporting has no current role in standard of care of TNBC and should be reported for research purposes only. If reported, it should be according to the International Working Group Criteria, 2014, on the stromal +/− intra-tumoural immune cells.	44%	II	A
Ki67 has no evident role in the current standard of care of TNBC and should be reported for research purposes only.	70%	II	A
It is mandatory to repeat hormonal receptors and HER2 assessment after neo-adjuvant treatment in TNBC patients with any residual disease. Capturing any overexpression of these markers avoids missing any opportunity of adjuvant therapy.	**75%**	III	A
If HER2 assessment by IHC changed to HER2+ after neo-adjuvant treatment in TNBC, it is preferred to offer adjuvant anti-HER2 therapy.	**92.6%**	Expert opinion	A
For early TNBC cases (cT1–2N0), mastectomy is not the preferred surgery for the ipsilateral breast, if the patient is eligible for BCT.	**100%**	IV	A
For *gBRCA*1/2 mutant early TNBC, the surgical option for the ipsilateral breast is controversial, if the patient is eligible for BCT.	**100%**	IV	C
For *gBRCA*1/2 mutant early TNBC patients, risk reduction contralateral mastectomy is advised. Taking into account, the patients’ age, stage, preference and risk of developing contralateral BC, rather than doctor’s recommendation solely.	69%	IV	C
TNBC biology per se is not an absolute indication for postoperative radiation therapy after mastectomy for all pT1–2 N0 TNBC cases.	**91.6%**	IV	C
Hypo-fractionation regimens can be considered for both early and advanced TNBC cases.	**87%**	II	B
It is preferred to offer radiation therapy boost to tumour bed for all cases of TNBC after lumpectomy.	58%	IV	B
After upfront surgery for pT1–3 N0 TNBC, offering regional nodal irradiation is based on the clinico-pathological features including but not limited to TNBC biology only, such as LVI and high grade.	**86%**	II	B
After mastectomy for pN1 (1–3 + LNs) TNBC, regional nodal irradiation is preferred.	**76%**	I	B
The preferred local management for the axilla for TNBC patients with Z-0011 criteria is controversial.	**100%**	II	B
There is no preference regarding the timing of reconstruction (immediate vs. delayed) after breast surgery for TNBC.	68%	II	B
For early-stage TNBC (cT2–3 N0); NAT is preferred over upfront surgery (regardless of the planned surgery type).	**93%**	II	A
For early-stage TNBC (cT2–3 N1); NAT is preferred over upfront surgery (regardless of the planned surgery type).	**100%**	I	A
NAT regimens for TNBC are preferred to be administered for 6–8 cycles.	**76%**	I	B
Adding platinum to the standard anthracyclines/taxanes NAT regimen is preferred especially with stage II–III TNBC and if suboptimal tumour response was achieved following anthracyclines.	**77.6%**	II	B
De-escalating the neo-adjuvant chemotherapy in TNBC by offering a short, anthracycline-free, taxane/platinum regimen only, for early responders (response adapted approach, ADAPT-TN trial), is not preferred.	**86%**	II	B
A carboplatin-including regimen is preferred in the NAT setting for *gBRCA*1/2 mutant TNBC.	69.2%	II	C
A carboplatin-including regimen is not preferred in the NAT setting for *wBRCA*1/2 TNBC	66.6%	II	C
No mature data yet to support the use of Pembrolizumab or Atezolizumab in the NAT setting for early TNBC.	72%	II	B
Capecitabine (6–8 cycles) is the preferred adjuvant therapy in case of absence of pCR after NAT (anthracyclines/taxanes) for TNBC.	**85%**	II	A
In the presence of pCR after NAT (anthracylines/taxanes) for TNBC, no further adjuvant systemic therapy is advised.	**93%**	II	A
Adjuvant chemotherapy for pT1a N0 TNBC can be omitted safely.	**88%**	II	A
Adjuvant chemotherapy for pT1b N0 TNBC is preferred.	**97%**	II	A
After upfront surgery, for stage I TNBC, the preferred adjuvant regimen is 6 cycles anthracyclines/taxanes.	**80%**	I	A
After upfront surgery, for stage II–III TNBC, the preferred adjuvant regimen is 6–8 cycles anthracyclines/taxanes.	61%	I	B
Dose dense AC-T regimen is a preferred one over standard regimen (/3 wks) in the adjuvant setting for stage II–III TNBC.	**88%**	II	A
If TNBC case developed metastasis, tissue biopsy and testing for ER, PR, HER2, PDL-1/germline *BRCA* mutations are recommended.	**95%**	I	A
After complete resection of isolated loco-regional recurrence (LRR) in non-metastatic TNBC, chemotherapy is recommended for 3–6 months.	**85%**	II	A
Metastatic TNBC disease (mTNBC) is a heterogeneous disease and should be categorized as the following: (1) PD-L1+ mTNBC, (2) *gBRCA* mutant mTNBC, (3) PD-L1−/w*BRCA* mTNBC or no testing done.	**89%**	I	A
Atezolizumab + nab-paclitaxel or Pembrolizumab + chemotherapy are preferred options over standard chemotherapy in the first-line setting for unresectable locally advanced/mTNBC expressing PD-L1 (PDL-1 ≥ 1% for Atezolizumab or CPS ≥ 10 for Pembrolizumab).	**86%**	I	B
For the 1^st^-line immunotherapy for PDL1+ mTNBC, no preference for one over the other (Atezolizumab/Pembrolizumab) as no head-to-head comparison.	58%	I	B
No mature data yet to support the use of Pembrolizumab in the subsequent lines in the metastatic setting of TNBC.	**93%**	II	B
Atezolizumab plus nab-paclitaxel should be offered for unresectable locally advanced/mTNBC patients after PD-L1 testing to identify the PD-L1+ population.	**93%**	I	A
If Atezolizumab is planned, PD-L1 should be tested using the Ventana SP142 IHC on the tumoural immune cells, with the cut-off value for PD-L1 positivity is 1%.	**94%**	I	A
If Pembrolizumab is planned, PD-L1 should be tested by 22C3 pharmDx test, with the cut-off of CPS ≥ 10.	**93%**	I	A
For *gBRCA*1/2 mutant mTNBC, a platinum or PARP inhibitor should be incorporated early in the treatment course.	66%	I	A
No mature data yet to support the use of Veliparib in the metastatic setting of TNBC.	**86%**	II	C
For mTNBC with PD-L1−/w*BRCA* or no testing done, the most efficacious sequencing of chemotherapy agents has yet to be defined.	**75%**	II	B
For mTNBC with PD-L1−/w*BRCA* or no testing done, paclitaxel or nab-paclitaxel/carboplatin are preferred as a combination regimen.	60%	III	C
For mTNBC with PD-L1−/w*BRCA* or no testing done, no preferred chemotherapy regimen in subsequent lines and enrolment in clinical trials is encouraged.	**75%**	III	B
For mTNBC with PD-L1−/w*BRCA* or no testing done, eribulin is a preferred subsequent line of therapy after prior anthracyclines and taxanes.	**75%**	II	B
In AR+ mTNBC, AR-directed therapy is not recommended for the management of mTNBC outside clinical trials.	**79%**	II	D
Sacituzumab govitecan is preferred over chemotherapy after ≥ 2 prior chemotherapies for mTNBC.	**86%**	I	A

## Data Availability

Not applicable.

## References

[1] Loblaw D.A., Prestrud A.A., Somerfield M.R., Oliver T.K., Brouwers M.C., Nam R.K., Lyman G.H., Basch E. (2012). American Society of Clinical Oncology Clinical Practice Guidelines: Formal systematic review-based consensus methodology. J. Clin. Oncol..

[2] Dykewicz C.A. (2001). Summary of the Guidelines for Preventing Opportunistic Infections among Hematopoietic Stem Cell Transplant Recipients. Clin. Infect. Dis..

[3] Penault-Llorca F., Viale G. (2012). Pathological and molecular diagnosis of triple-negative breast cancer: A clinical perspective. Ann. Oncol..

[4] Verma S., Provencher L., Dent R. (2011). Emerging trends in the treatment of triple-negative breast cancer in Canada: A survey. Curr. Oncol..

[5] Swedish National Guidelines, Breast Cancer. https://www.swebcg.se/wp-content/uploads/2016/10/nationellt-vardprogram-brostcancer_200211.pdf.

[6] Allison K.H., Hammond M.E.H., Dowsett M., McKernin S.E., Carey L.A., Fitzgibbons P.L., Hayes D.F., Lakhani S.R., Chavez-MacGregor M., Perlmutter J. (2020). Estrogen and Progesterone Receptor Testing in Breast Cancer: American Society of Clinical Oncology/College of American Pathologists Guideline Update. Arch. Pathol. Lab. Med..

[7] Fei F., Siegal G.P., Wei S. (2021). Characterization of estrogen receptor-low-positive breast cancer. Breast Cancer Res. Treat..

[8] Iwamoto T., Booser D., Valero V., Murray J.L., Koenig K., Esteva F.J., Ueno N.T., Zhang J., Shi W., Qi Y. (2012). Estrogen receptor (ER) mRNA and ER-related gene expression in breast cancers that are 1% to 10% ER-positive by immunohistochemistry. J. Clin. Oncol..

[9] Fujii T., Kogawa T., Dong W., Sahin A.A., Moulder S., Litton J.K., Tripathy D., Iwamoto T., Hunt K.K., Pusztai L. (2017). Revisiting the definition of estrogen receptor positivity in HER2-negative primary breast cancer. Ann. Oncol..

[10] Landmann A., Farrugia D.J., Zhu L., Diego E.J., Johnson R.R., Soran A., Dabbs D.J., Clark B.Z., Puhalla S.L., Jankowitz R.C. (2018). Low Estrogen Receptor (ER)–Positive Breast Cancer and Neoadjuvant Systemic Chemotherapy: Is Response Similar to Typical ER-Positive or ER-Negative Disease?. Am. J. Clin. Pathol..

[11] Prabhu J.S., Korlimarla A., Desai K., Alexander A., Raghavan R., Anupama C., Dendukuri N., Manjunath S., Correa M., Raman N. (2014). A Majority of Low (1–10%) ER Positive Breast Cancers Behave Like Hormone Receptor Negative Tumors. J. Cancer.

[12] Yi M., Huo L., Koenig K.B., Mittendorf E.A., Meric-Bernstam F., Kuerer H.M., Bedrosian I., Buzdar A.U., Symmans W.F., Crow J.R. (2014). Which threshold for ER positivity? A retrospective study based on 9639 patients. Ann. Oncol..

[13] Lovejoy L.A., Turner C.E., Wells J.M., Shriver C.D., Ellsworth R.E. (2020). Heritability of Low ER Staining/HER2-Breast Tumors: Are We Missing an Opportunity for Germline Testing?. Genes.

[14] Balic M., Thomssen C., Würstlein R., Gnant M., Harbeck N. (2019). St. Gallen/Vienna 2019: A Brief Summary of the Consensus Discussion on the Optimal Primary Breast Cancer Treatment. Breast Care.

[15] Coates A.S., Winer E.P., Goldhirsch A., Gelber R.D., Gnant M., Piccart-Gebhart M., Thürlimann B., Senn H.J. (2015). Tailoring therapies—Improving the management of early breast cancer: St Gallen International Expert Consensus on the Primary Therapy of Early Breast Cancer 2015. Ann. Oncol. Off. J. Eur. Soc. Med. Oncol..

[16] Thomssen C., Balic M., Harbeck N., Gnant M. (2021). St. Gallen/Vienna 2021: A Brief Summary of the Consensus Discussion on Customizing Therapies for Women with Early Breast Cancer. Breast Care.

[17] Chen H., Wu J., Zhang Z., Tang Y., Li X., Liu S., Cao S., Li X. (2018). Association between BRCA Status and Triple-Negative Breast Cancer: A Meta-Analysis. Front. Pharmacol..

[18] Copson E.R., Maishman T.C., Tapper W.J., Cutress R.I., Greville-Heygate S., Altman D.G., Eccles B., Gerty S., Durcan L.T., Jones L. (2018). Germline BRCA mutation and outcome in young-onset breast cancer (POSH): A prospective cohort study. Lancet Oncol..

[19] Engel C., Rhiem K., Hahnen E., Loibl S., Weber K.E., Seiler S., Zachariae S., Hauke J., Wappenschmidt B., Waha A. (2018). Prevalence of pathogenic BRCA1/2 germline mutations among 802 women with unilateral triple-negative breast cancer without family cancer history. BMC Cancer.

[20] Armstrong N., Ryder S., Forbes C., Ross J., Quek R.G. (2019). A systematic review of the international prevalence of BRCA mutation in breast cancer. Clin. Epidemiol..

[21] Daly M.B., Pal T., Berry M.P., Buys S.S., Dickson P., Domchek S.M., Elkhanany A., Friedman S., Goggins M., Hutton M.L. (2021). Genetic/Familial High-Risk Assessment: Breast, Ovarian, and Pancreatic, Version 2.2021, NCCN Clinical Practice Guidelines in Oncology. J. Natl. Compr. Cancer Netw..

[22] Bonnefoi H., Grellety T., Tredan O., Saghatchian M., Dalenc F., Mailliez A., L’Haridon T., Cottu P., Abadie-Lacourtoisie S., You B. (2016). A phase II trial of abiraterone acetate plus prednisone in patients with triple-negative androgen receptor positive locally advanced or metastatic breast cancer (UCBG 12-1). Ann. Oncol. Off. J. Eur. Soc. Med. Oncol..

[23] Gucalp A., Tolaney S., Isakoff S.J., Ingle J.N., Liu M.C., Carey L.A., Blackwell K., Rugo H., Nabell L., Forero A. (2013). Phase II trial of bicalutamide in patients with androgen receptor-positive, estrogen receptor-negative metastatic Breast Cancer. Clin. Cancer Res. Off. J. Am. Assoc. Cancer Res..

[24] Traina T.A., Miller K., Yardley D.A., Eakle J., Schwartzberg L.S., O’Shaughnessy J., Gradishar W., Schmid P., Winer E., Kelly C. (2018). Enzalutamide for the Treatment of Androgen Receptor-Expressing Triple-Negative Breast Cancer. J. Clin. Oncol. Off. J. Am. Soc. Clin. Oncol..

[25] Lehmann B.D., Bauer J.A., Chen X., Sanders M.E., Chakravarthy A.B., Shyr Y., Pietenpol J.A. (2011). Identification of human triple-negative breast cancer subtypes and preclinical models for selection of targeted therapies. J. Clin. Investig..

[26] Dieci M.V., Tsvetkova V., Griguolo G., Miglietta F., Mantiero M., Tasca G., Cumerlato E., Giorgi C.A., Giarratano T., Faggioni G. (2019). Androgen Receptor Expression and Association with Distant Disease-Free Survival in Triple Negative Breast Cancer: Analysis of 263 Patients Treated with Standard Therapy for Stage I–III Disease. Front. Oncol..

[27] Bhattarai S., Klimov S., Mittal K., Krishnamurti U., Li X.B., Oprea-Ilies G., Wetherilt C.S., Riaz A., Aleskandarany M.A., Green A.R. (2019). Prognostic Role of Androgen Receptor in Triple Negative Breast Cancer: A Multi-Institutional Study. Cancers.

[28] Anestis A., Zoi I., Papavassiliou A.G., Karamouzis M.V. (2020). Androgen Receptor in Breast Cancer-Clinical and Preclinical Research Insights. Molecules.

[29] ClinicalTrials.gov. M.D. Anderson Cancer Center—Enzalutamide and Paclitaxel Before Surgery in Treating Patients With Stage I-III Androgen Receptor-Positive Triple-Negative Breast Cancer—ClinicalTrials.gov Identifier: NCT02689427. NCT02689427.

[30] ClinicalTrials.gov Memorial Sloan Kettering Cancer Center—Bicalutamide in Treating Patients with Metastatic Breast Cancer—ClinicalTrials.gov Identifier: NCT00468715. NCT00468715.

[31] Denkert C., von Minckwitz G., Darb-Esfahani S., Lederer B., Heppner B.I., Weber K.E., Budczies J., Huober J., Klauschen F., Furlanetto J. (2018). Tumour-infiltrating lymphocytes and prognosis in different subtypes of breast cancer: A pooled analysis of 3771 patients treated with neoadjuvant therapy. Lancet Oncol..

[32] Dieci M.V., Mathieu M.C., Guarneri V., Conte P., Delaloge S., Andre F., Goubar A. (2015). Prognostic and predictive value of tumor-infiltrating lymphocytes in two phase III randomized adjuvant breast cancer trials. Ann. Oncol. Off. J. Eur. Soc. Med. Oncol..

[33] Gao G., Wang Z., Qu X., Zhang Z. (2020). Prognostic value of tumor-infiltrating lymphocytes in patients with triple-negative breast cancer: A systematic review and meta-analysis. BMC Cancer.

[34] Loi S., Drubay D., Adams S., Pruneri G., Francis P.A., Lacroix-Triki M., Joensuu H., Dieci M.V., Badve S., Demaria S. (2019). Tumor-Infiltrating Lymphocytes and Prognosis: A Pooled Individual Patient Analysis of Early-Stage Triple-Negative Breast Cancers. J. Clin. Oncol..

[35] Asano Y., Kashiwagi S., Goto W., Takada K., Takahashi K., Hatano T., Takashima T., Tomita S., Motomura H., Ohsawa M. (2018). Prediction of Treatment Response to Neoadjuvant Chemotherapy in Breast Cancer by Subtype Using Tumor-infiltrating Lymphocytes. Anticancer Res..

[36] Loi S., Sirtaine N., Piette F., Salgado R., Viale G., Van Eenoo F., Rouas G., Francis P., Crown J.P.A., Hitre E. (2013). Prognostic and Predictive Value of Tumor-Infiltrating Lymphocytes in a Phase III Randomized Adjuvant Breast Cancer Trial in Node-Positive Breast Cancer Comparing the Addition of Docetaxel to Doxorubicin with Doxorubicin-Based Chemotherapy: BIG 02-98. J. Clin. Oncol..

[37] Badr N.M., Berditchevski F., Shaaban A.M. (2020). The Immune Microenvironment in Breast Carcinoma: Predictive and Prognostic Role in the Neoadjuvant Setting. Pathobiology.

[38] Denkert C., Loibl S., Noske A., Roller M., Müller B.M., Komor M., Budczies J., Darb-Esfahani S., Kronenwett R., Hanusch C. (2009). Tumor-Associated Lymphocytes as an Independent Predictor of Response to Neoadjuvant Chemotherapy in Breast Cancer. J. Clin. Oncol..

[39] Salgado R., Denkert C., Demaria S., Sirtaine N., Klauschen F., Pruneri G., Wienert S., Eynden G.V.D., Baehner F.L., Penault-Llorca F. (2015). The evaluation of tumor-infiltrating lymphocytes (TILs) in breast cancer: Recommendations by an International TILs Working Group 2014. Ann. Oncol..

[40] Gonzalez-Cortijo L., Murillo R., Acevedo A., Sainz de la Cuesta R., Hernandez-Cortes G., Martinez de Vega V., Linares S., Perez-Carrion R., Gonzalez F., Rodriguez-Marquez C. (2016). Association between distinct intratumoral and stromal tumor infiltrating immune cells and pathologic complete response (pCR) in different breast cancer subtypes after neoadjuvant chemotherapy (NAC). J. Clin. Oncol..

[41] Khoury T., Nagrale V., Opyrchal M., Peng X., Wang D., Yao S. (2018). Prognostic Significance of Stromal Versus Intratumoral Infiltrating Lymphocytes in Different Subtypes of Breast Cancer Treated with Cytotoxic Neoadjuvant Chemotherapy. Appl. Immunohistochem. Mol. Morphol. AIMM.

[42] Pathmanathan N., Balleine R.L. (2013). Ki67 and proliferation in breast cancer. J. Clin. Pathol..

[43] Nielsen T.O., Leung S.C.Y., Rimm D.L., Dodson A., Acs B., Badve S., Denkert C., Ellis M.J., Fineberg S., Flowers M. (2020). Assessment of Ki67 in Breast Cancer: Updated Recommendations from the International Ki67 in Breast Cancer Working Group. J. Natl. Cancer Inst..

[44] Cardoso F., Kyriakides S., Ohno S., Penault-Llorca F., Poortmans P., Rubio I.T., Zackrisson S., Senkus E. (2019). Early breast cancer: ESMO Clinical Practice Guidelines for diagnosis, treatment and follow-up. Ann. Oncol..

[45] Wu Q., Ma G., Deng Y., Luo W., Zhao Y., Li W., Zhou Q. (2019). Prognostic Value of Ki-67 in Patients with Resected Triple-Negative Breast Cancer: A Meta-Analysis. Front. Oncol..

[46] Zhu X., Chen L., Huang B., Wang Y., Ji L., Wu J., Di G., Liu G., Yu K., Shao Z. (2020). The prognostic and predictive potential of Ki-67 in triple-negative breast cancer. Sci. Rep..

[47] Provenzano E., Bossuyt V., Viale G., Cameron D., Badve S., Denkert C., MacGrogan G., Penault-Llorca F., Boughey J., Curigliano G. (2015). Standardization of pathologic evaluation and reporting of postneoadjuvant specimens in clinical trials of breast cancer: Recommendations from an international working group. Mod. Pathol. Off. J. United States Can. Acad. Pathol. Inc..

[48] Li C., Fan H., Xiang Q., Xu L., Zhang Z., Liu Q., Zhang T., Ling J., Zhou Y., Zhao X. (2019). Prognostic value of receptor status conversion following neoadjuvant chemotherapy in breast cancer patients: A systematic review and meta-analysis. Breast Cancer Res. Treat..

[49] Abdulkarim B.S., Cuartero J., Hanson J., Deschênes J., Lesniak D., Sabri S. (2011). Increased risk of locoregional recurrence for women with T1-2N0 triple-negative breast cancer treated with modified radical mastectomy without adjuvant radiation therapy compared with breast-conserving therapy. J. Clin. Oncol. Off. J. Am. Soc. Clin. Oncol..

[50] Bhoo-Pathy N., Verkooijen H.M., Wong F.-Y., Pignol J.-P., Kwong A., Tan E.-Y., Aishah Taib N., Nei W.-L., Ho G.-F., Tan B. (2015). Prognostic role of adjuvant radiotherapy in triple-negative breast cancer: A historical cohort study. Int. J. Cancer.

[51] Zumsteg Z.S., Morrow M., Arnold B., Zheng J., Zhang Z., Robson M., Traina T., McCormick B., Powell S., Ho A.Y. (2013). Breast-conserving therapy achieves locoregional outcomes comparable to mastectomy in women with T1-2N0 triple-negative breast cancer. Ann. Surg. Oncol..

[52] Li H., Chen Y., Wang X., Tang L., Guan X. (2019). T1-2N0M0 Triple-Negative Breast Cancer Treated with Breast-Conserving Therapy Has Better Survival Compared to Mastectomy: A SEER Population-Based Retrospective Analysis. Clin. Breast Cancer.

[53] Wang J., Xie X., Wang X., Tang J., Pan Q., Zhang Y., Di M. (2013). Locoregional and distant recurrences after breast conserving therapy in patients with triple-negative breast cancer: A meta-analysis. Surg. Oncol..

[54] Pierce L.J., Phillips K.A., Griffith K.A., Buys S., Gaffney D.K., Moran M.S., Haffty B.G., Ben-David M., Kaufman B., Garber J.E. (2010). Local therapy in BRCA1 and BRCA2 mutation carriers with operable breast cancer: Comparison of breast conservation and mastectomy. Breast Cancer Res. Treat..

[55] Pierce L.J., Levin A.M., Rebbeck T.R., Ben-David M.A., Friedman E., Solin L.J., Harris E.E., Gaffney D.K., Haffty B.G., Dawson L.A. (2006). Ten-year multi-institutional results of breast-conserving surgery and radiotherapy in BRCA1/2-associated stage I/II breast cancer. J. Clin. Oncol. Off. J. Am. Soc. Clin. Oncol..

[56] Valachis A., Nearchou A.D., Lind P. (2014). Surgical management of breast cancer in BRCA-mutation carriers: A systematic review and meta-analysis. Breast Cancer Res. Treat..

[57] Co M., Liu T., Leung J., Li C.H., Tse T., Wong M., Kwong A. (2020). Breast Conserving Surgery for BRCA Mutation Carriers—A Systematic Review. Clin. Breast Cancer.

[58] Omranipour R., Bobin J.Y., Esouyeh M. (2008). Skin Sparing Mastectomy and Immediate Breast Reconstruction (SSMIR) for early breast cancer: Eight years single institution experience. World J. Surg. Oncol..

[59] Chiba A., Hoskin T.L., Hallberg E.J., Cogswell J.A., Heins C.N., Couch F.J., Boughey J.C. (2016). Impact that Timing of Genetic Mutation Diagnosis has on Surgical Decision Making and Outcome for BRCA1/BRCA2 Mutation Carriers with Breast Cancer. Ann. Surg. Oncol..

[60] McGuire K.P., Mamounas E.P. (2020). Management of Hereditary Breast Cancer: ASCO, ASTRO, and SSO Guideline. Ann. Surg. Oncol..

[61] van den Broek A.J., van’t Veer L.J., Hooning M.J., Cornelissen S., Broeks A., Rutgers E.J., Smit V.T.H.B.M., Cornelisse C.J., van Beek M., Janssen-Heijnen M.L. (2016). Impact of Age at Primary Breast Cancer on Contralateral Breast Cancer Risk in BRCA1/2 Mutation Carriers. J. Clin. Oncol. Off. J. Am. Soc. Clin. Oncol..

[62] Evans D.G.R., Ingham S.L., Baildam A., Ross G.L., Lalloo F., Buchan I., Howell A. (2013). Contralateral mastectomy improves survival in women with BRCA1/2-associated breast cancer. Breast Cancer Res. Treat..

[63] Heemskerk-Gerritsen B.A.M., Rookus M.A., Aalfs C.M., Ausems M.G.E.M., Collée J.M., Jansen L., Kets C.M., Keymeulen K.B.M.I., Koppert L.B., Meijers-Heijboer H.E.J. (2015). Improved overall survival after contralateral risk-reducing mastectomy in BRCA1/2 mutation carriers with a history of unilateral breast cancer: A prospective analysis. Int. J. Cancer.

[64] Metcalfe K., Gershman S., Ghadirian P., Lynch H.T., Snyder C., Tung N., Kim-Sing C., Eisen A., Foulkes W.D., Rosen B. (2014). Contralateral mastectomy and survival after breast cancer in carriers of BRCA1 and BRCA2 mutations: Retrospective analysis. BMJ.

[65] Haque W., Verma V., Farach A., Brian Butler E., Teh B.S. (2019). Postmastectomy radiation therapy for triple negative, node-negative breast cancer. Radiother. Oncol. J. Eur. Soc. Ther. Radiol. Oncol..

[66] Jagsi R., Raad R.A., Goldberg S., Sullivan T., Michaelson J., Powell S.N., Taghian A.G. (2005). Locoregional recurrence rates and prognostic factors for failure in node-negative patients treated with mastectomy: Implications for postmastectomy radiation. Int. J. Radiat. Oncol. Biol. Phys..

[67] O’Rorke M.A., Murray L.J., Brand J.S., Bhoo-Pathy N. (2016). The value of adjuvant radiotherapy on survival and recurrence in triple-negative breast cancer: A systematic review and meta-analysis of 5507 patients. Cancer Treat. Rev..

[68] Truong P.T., Lesperance M., Culhaci A., Kader H.A., Speers C.H., Olivotto I.A. (2005). Patient subsets with T1-T2, node-negative breast cancer at high locoregional recurrence risk after mastectomy. Int. J. Radiat. Oncol. Biol. Phys..

[69] Yao Y., Chu Y., Xu B., Hu Q., Song Q. (2019). Radiotherapy after surgery has significant survival benefits for patients with triple-negative breast cancer. Cancer Med..

[70] Chen X., Yu X., Chen J., Yang Z., Shao Z., Zhang Z., Guo X., Feng Y. (2013). Radiotherapy can improve the disease-free survival rate in triple-negative breast cancer patients with T1-T2 disease and one to three positive lymph nodes after mastectomy. Oncologist.

[71] Yildirim E., Berberoglu U. (2007). Can a subgroup of node-negative breast carcinoma patients with T1-2 tumor who may benefit from postmastectomy radiotherapy be identified?. Int. J. Radiat. Oncol. Biol. Phys..

[72] Smith B.D., Bellon J.R., Blitzblau R., Freedman G., Haffty B., Hahn C., Halberg F., Hoffman K., Horst K., Moran J. (2018). Radiation therapy for the whole breast: Executive summary of an American Society for Radiation Oncology (ASTRO) evidence-based guideline. Pract. Radiat. Oncol..

[73] Bane A.L., Whelan T.J., Pond G.R., Parpia S., Gohla G., Fyles A.W., Pignol J.P., Pritchard K.I., Chambers S., Levine M.N. (2014). Tumor factors predictive of response to hypofractionated radiotherapy in a randomized trial following breast conserving therapy. Ann. Oncol..

[74] Moran M.S. (2015). Radiation therapy in the locoregional treatment of triple-negative breast cancer. Lancet Oncol..

[75] Sioshansi S., Ehdaivand S., Cramer C., Lomme M.M., Price L.L., Wazer D.E. (2012). Triple negative breast cancer is associated with an increased risk of residual invasive carcinoma after lumpectomy. Cancer.

[76] Jones H.A., Antonini N., Hart A.A., Peterse J.L., Horiot J.C., Collin F., Poortmans P.M., Oei S.B., Collette L., Struikmans H. (2009). Impact of pathological characteristics on local relapse after breast-conserving therapy: A subgroup analysis of the EORTC boost versus no boost trial. J. Clin. Oncol..

[77] National Institute for Health and Care Excellence (NICE) Early and Locally Advanced Breast Cancer: Diagnosis and Management. https://www.nice.org.uk/guidance/ng101.

[78] Recht A., Comen E.A., Fine R.E., Fleming G.F., Hardenbergh P.H., Ho A.Y., Hudis C.A., Hwang E.S., Kirshner J.J., Morrow M. (2016). Postmastectomy Radiotherapy: An American Society of Clinical Oncology, American Society for Radiation Oncology, and Society of Surgical Oncology Focused Guideline Update. J. Clin. Oncol..

[79] Whelan T.J., Olivotto I.A., Parulekar W.R., Ackerman I., Chua B.H., Nabid A., Vallis K.A., White J.R., Rousseau P., Fortin A. (2015). Regional Nodal Irradiation in Early-Stage Breast Cancer. N. Engl. J. Med..

[80] Giuliano A.E., Ballman K.V., McCall L., Beitsch P.D., Brennan M.B., Kelemen P.R., Ollila D.W., Hansen N.M., Whitworth P.W., Blumencranz P.W. (2017). Effect of Axillary Dissection vs No Axillary Dissection on 10-Year Overall Survival among Women with Invasive Breast Cancer and Sentinel Node Metastasis. JAMA.

[81] Morrow M., Mehrara B. (2009). Prophylactic mastectomy and the timing of breast reconstruction. Br. J. Surg..

[82] Yoon A.P., Qi J., Brown D.L., Kim H.M., Hamill J.B., Erdmann-Sager J., Pusic A.L., Wilkins E.G. (2018). Outcomes of immediate versus delayed breast reconstruction: Results of a multicenter prospective study. Breast.

[83] Dubsky P., Pinker K., Cardoso F., Montagna G., Ritter M., Denkert C., Rubio I.T., de Azambuja E., Curigliano G., Gentilini O. (2021). Breast conservation and axillary management after primary systemic therapy in patients with early-stage breast cancer: The Lucerne toolbox. Lancet Oncol..

[84] Cortazar P., Zhang L., Untch M., Mehta K., Costantino J.P., Wolmark N., Bonnefoi H., Cameron D., Gianni L., Valagussa P. (2014). Pathological complete response and long-term clinical benefit in breast cancer: The CTNeoBC pooled analysis. Lancet.

[85] von Minckwitz G., Untch M., Blohmer J.-U., Costa S.D., Eidtmann H., Fasching P.A., Gerber B., Eiermann W., Hilfrich J., Huober J. (2012). Definition and impact of pathologic complete response on prognosis after neoadjuvant chemotherapy in various intrinsic breast cancer subtypes. J. Clin. Oncol. Off. J. Am. Soc. Clin. Oncol..

[86] Xia L.-Y., Hu Q.-L., Zhang J., Xu W.-Y., Li X.-S. (2020). Survival outcomes of neoadjuvant versus adjuvant chemotherapy in triple-negative breast cancer: A meta-analysis of 36,480 cases. World J. Surg. Oncol..

[87] Bagegni N.A., Tao Y., Ademuyiwa F.O. (2019). Clinical outcomes with neoadjuvant versus adjuvant chemotherapy for triple negative breast cancer: A report from the National Cancer Database. PLoS ONE.

[88] Masuda N., Lee S.-J., Ohtani S., Im Y.-H., Lee E.-S., Yokota I., Kuroi K., Im S.-A., Park B.-W., Kim S.-B. (2017). Adjuvant Capecitabine for Breast Cancer after Preoperative Chemotherapy. N. Engl. J. Med..

[89] Biswas T., Efird J.T., Prasad S., Jindal C., Walker P.R. (2017). The survival benefit of neoadjuvant chemotherapy and pCR among patients with advanced stage triple negative breast cancer. Oncotarget.

[90] Gamucci T., Pizzuti L., Sperduti I., Mentuccia L., Vaccaro A., Moscetti L., Marchetti P., Carbognin L., Michelotti A., Iezzi L. (2018). Neoadjuvant chemotherapy in triple-negative breast cancer: A multicentric retrospective observational study in real-life setting. J. Cell. Physiol..

[91] Sikov W.M., Berry D.A., Perou C.M., Singh B., Cirrincione C.T., Tolaney S.M., Kuzma C.S., Pluard T.J., Somlo G., Port E.R. (2015). Impact of the addition of carboplatin and/or bevacizumab to neoadjuvant once-per-week paclitaxel followed by dose-dense doxorubicin and cyclophosphamide on pathologic complete response rates in stage II to III triple-negative breast cancer: CALGB 40603 (Alliance). J. Clin. Oncol. Off. J. Am. Soc. Clin. Oncol..

[92] Loibl S., Weber K.E., Timms K.M., Elkin E.P., Hahnen E., Fasching P.A., Lederer B., Denkert C., Schneeweiss A., Braun S. (2018). Survival analysis of carboplatin added to an anthracycline/taxane-based neoadjuvant chemotherapy and HRD score as predictor of response—final results from GeparSixto. Ann. Oncol..

[93] Loibl S., O’Shaughnessy J., Untch M., Sikov W.M., Rugo H.S., McKee M.D., Huober J., Golshan M., von Minckwitz G., Maag D. (2018). Addition of the PARP inhibitor veliparib plus carboplatin or carboplatin alone to standard neoadjuvant chemotherapy in triple-negative breast cancer (BrighTNess): A randomised, phase 3 trial. Lancet Oncol..

[94] Pandy J.G.P., Balolong-Garcia J.C., Cruz-Ordinario M.V.B., Que F.V.F. (2019). Triple negative breast cancer and platinum-based systemic treatment: A meta-analysis and systematic review. BMC Cancer.

[95] Gluz O., Nitz U., Liedtke C., Christgen M., Grischke E.-M., Forstbauer H., Braun M., Warm M., Hackmann J., Uleer C. (2018). Comparison of neoadjuvant nab-paclitaxel+ carboplatin vs nab-paclitaxel+ gemcitabine in triple-negative breast cancer: Randomized WSG-ADAPT-TN trial results. JNCI J. Natl. Cancer Inst..

[96] Caramelo O., Silva C., Caramelo F., Frutuoso C., Almeida-Santos T. (2019). The effect of neoadjuvant platinum-based chemotherapy in BRCA mutated triple negative breast cancers -systematic review and meta-analysis. Hered. Cancer Clin. Pract..

[97] Hoadley K.A., Powell B.C., Kanavy D., Marron D., Mose L.E., Hyslop T., Berry D.A., Hahn O., Tolaney S.M., Sikov W.M. (2020). Abstract P4-05-03: Mutational analysis of triple-negative breast cancer (TNBC): CALGB 40603 (Alliance). Cancer Res..

[98] Kim G.M., Jeung H.-C., Jung K.H., Kim S.H., Kim H.J., Lee K.H., Park K.H., Lee J.E., Ahn M.S., Kohn S. (2017). PEARLY: A randomized, multicenter, open-label, phase III trial comparing anthracyclines followed by taxane versus anthracyclines followed by taxane plus carboplatin as (neo)adjuvant therapy in patients with early triple-negative breast cancer. J. Clin. Oncol..

[99] Schmid P., Cortes J., Pusztai L., McArthur H., Kümmel S., Bergh J., Denkert C., Park Y.H., Hui R., Harbeck N. (2020). Pembrolizumab for Early Triple-Negative Breast Cancer. N. Engl. J. Med..

[100] Mittendorf E.A., Zhang H., Barrios C.H., Saji S., Jung K.H., Hegg R., Koehler A., Sohn J., Iwata H., Telli M.L. (2020). Neoadjuvant atezolizumab in combination with sequential nab-paclitaxel and anthracycline-based chemotherapy versus placebo and chemotherapy in patients with early-stage triple-negative breast cancer (IMpassion031): A randomised, double-blind, phase 3 trial. Lancet.

[101] Gianni L., Huang C.-S., Egle D., Bermejo B., Zamagni C., Thill M., Anton A., Zambelli S., Bianchini G., Russo S. (2020). Abstract GS3-04: Pathologic complete response (pCR) to neoadjuvant treatment with or without atezolizumab in triple negative, early high-risk and locally advanced breast cancer. NeoTRIPaPDL1 Michelangelo randomized study. Cancer Res..

[102] ClinicalTrials.gov ECOG-ACRIN Cancer Research Group—Platinum Based Chemotherapy or Capecitabine in Treating Patients with Residual Triple-Negative Basal-Like Breast Cancer Following Neoadjuvant Chemotherapy—ClinicalTrials.gov Identifier: NCT02445391. NCT02445391.

[103] Vaz-Luis I., Ottesen R.A., Hughes M.E., Mamet R., Burstein H.J., Edge S.B., Gonzalez-Angulo A.M., Moy B., Rugo H.S., Theriault R.L. (2014). Outcomes by tumor subtype and treatment pattern in women with small, node-negative breast cancer: A multi-institutional study. J. Clin. Oncol. Off. J. Am. Soc. Clin. Oncol..

[104] Du Z.-L., Wang Y., Wang D.-Y., Zhang L., Bian Z.-M., Deng Y., Xu C.-S., Lin D.-C., Xie L., Jia Y. (2020). Evaluation of a beneficial effect of adjuvant chemotherapy in patients with stage I triple-negative breast cancer: A population-based study using the SEER 18 database. Breast Cancer Res. Treat..

[105] An X., Lei X., Huang R., Luo R., Li H., Xu F., Yuan Z., Wang S., de Nonneville A., Gonçalves A. (2020). Adjuvant chemotherapy for small, lymph node–negative, triple-negative breast cancer: A single-center study and a meta-analysis of the published literature. Cancer.

[106] Henderson I.C., Berry D.A., Demetri G.D., Cirrincione C.T., Goldstein L.J., Martino S., Ingle J.N., Cooper M.R., Hayes D.F., Tkaczuk K.H. (2003). Improved outcomes from adding sequential Paclitaxel but not from escalating Doxorubicin dose in an adjuvant chemotherapy regimen for patients with node-positive primary breast cancer. J. Clin. Oncol. Off. J. Am. Soc. Clin. Oncol..

[107] Rodríguez-Lescure Á., Martín M., Ruiz A., Alba E., Calvo L., García-Asenjo J.L., Guitian M., de la Cruz A., Aranda I., de Álava E. (2007). Subgroup analysis of GEICAM 9906 trial comparing six cycles of FE90C (FEC) to four cycles of FE90C followed by 8 weekly paclitaxel administrations (FECP): Relevance of HER2 and hormonal status (HR). J. Clin. Oncol..

[108] Yu K.-D., Ye F.-G., He M., Fan L., Ma D., Mo M., Wu J., Liu G.-Y., Di G.-H., Zeng X.-H. (2020). Effect of adjuvant paclitaxel and carboplatin on survival in women with triple-negative breast cancer: A phase 3 randomized clinical trial. JAMA Oncol..

[109] Gray R., Bradley R., Braybrooke J., Liu Z., Peto R., Davies L., Dodwell D., McGale P., Pan H., Taylor C. (2019). Increasing the dose intensity of chemotherapy by more frequent administration or sequential scheduling: A patient-level meta-analysis of 37,298 women with early breast cancer in 26 randomised trials. Lancet.

[110] Amir E., Clemons M., Purdie C.A., Miller N., Quinlan P., Geddie W., Coleman R.E., Freedman O.C., Jordan L.B., Thompson A.M. (2012). Tissue confirmation of disease recurrence in breast cancer patients: Pooled analysis of multi-centre, multi-disciplinary prospective studies. Cancer Treat. Rev..

[111] Wapnir I.L., Price K.N., Anderson S.J., Robidoux A., Martín M., Nortier J.W.R., Paterson A.H.G., Rimawi M.F., Láng I., Baena-Cañada J.M. (2018). Efficacy of Chemotherapy for ER-Negative and ER-Positive Isolated Locoregional Recurrence of Breast Cancer: Final Analysis of the CALOR Trial. J. Clin. Oncol. Off. J. Am. Soc. Clin. Oncol..

[112] Food and Drug Administration (FDA) FDA Approves Atezolizumab for PD-L1 Positive Unresectable Locally Advanced or Metastatic Triple-Negative Breast Cancer. https://www.fda.gov/drugs/drug-approvals-and-databases/fda-approves-atezolizumab-pd-l1-positive-unresectable-locally-advanced-or-metastatic-triple-negative.

[113] Schmid P., Adams S., Rugo H.S., Schneeweiss A., Barrios C.H., Iwata H., Diéras V., Hegg R., Im S.-A., Shaw Wright G. (2018). Atezolizumab and Nab-Paclitaxel in Advanced Triple-Negative Breast Cancer. N. Engl. J. Med..

[114] Schmid P., Rugo H.S., Adams S., Schneeweiss A., Barrios C.H., Iwata H., Diéras V., Henschel V., Molinero L., Chui S.Y. (2020). Atezolizumab plus nab-paclitaxel as first-line treatment for unresectable, locally advanced or metastatic triple-negative breast cancer (IMpassion130): Updated efficacy results from a randomised, double-blind, placebo-controlled, phase 3 trial. Lancet Oncol..

[115] Emens L.A., Adams S., Barrios C.H., Dieras V.C., Iwata H., Loi S., Rugo H.S., Schneeweiss A., Winer E.P., Patel S. (2020). LBA16 IMpassion130: Final OS analysis from the pivotal phase III study of atezolizumab + nab-paclitaxel vs placebo + nab-paclitaxel in previously untreated locally advanced or metastatic triple-negative breast cancer. Ann. Oncol..

[116] Miles D., Gligorov J., Andre F., Cameron D., Schneeweiss A., Barrios C., Xu B., Wardley A.M., Kaen D., Andrade L. (2020). Primary results from IMpassion131, a double-blind placebo-controlled randomised phase III trial of first-line paclitaxel (PAC)+/−atezolizumab (atezo) for unresectable locally advanced/metastatic triple-negative breast cancer (mTNBC). Ann. Oncol..

[117] Cortes J., Cescon D.W., Rugo H.S., Nowecki Z., Im S.-A., Yusof M.M., Gallardo C., Lipatov O., Barrios C.H., Holgado E. (2020). Pembrolizumab plus chemotherapy versus placebo plus chemotherapy for previously untreated locally recurrent inoperable or metastatic triple-negative breast cancer (KEYNOTE-355): A randomised, placebo-controlled, double-blind, phase 3 clinical trial. Lancet.

[118] Cortés J., Lipatov O., Im S.-A., Gonçalves A., Lee K., Schmid P., Tamura K., Testa L., Witzel I., Ohtani S. (2019). KEYNOTE-119: Phase III study of pembrolizumab (pembro) versus single-agent chemotherapy (chemo) for metastatic triple negative breast cancer (mTNBC). Ann. Oncol..

[119] Vennapusa B., Baker B., Kowanetz M., Boone J., Menzl I., Bruey J.-M., Fine G., Mariathasan S., McCaffery I., Mocci S. (2019). Development of a PD-L1 Complementary Diagnostic Immunohistochemistry Assay (SP142) for Atezolizumab. Appl. Immunohistochem. Mol. Morphol. AIMM.

[120] Rugo H.S., Loi S., Adams S., Schmid P., Schneeweiss A., Barrios C.H., Iwata H., Dieras V., Winer E.P., Kockx M. (2019). Performance of PD-L1 immunohistochemistry (IHC) assays in unresectable locally advanced or metastatic triple-negative breast cancer (mTNBC): Post-hoc analysis of IMpassion130. Ann. Oncol..

[121] Kaya V., Yildirim M., Yazici G., Gunduz S., Bozcuk H., Paydas S. (2018). Effectiveness of platinum-based treatment for triple negative metastatic breast cancer: A meta-analysis. Asian Pac. J. Cancer Prev. APJCP.

[122] Azim H.A., Ghosn M., Oualla K., Kassem L. (2020). Personalized treatment in metastatic triple-negative breast cancer: The outlook in 2020. Breast J..

[123] Tutt A., Tovey H., Cheang M.C.U., Kernaghan S., Kilburn L., Gazinska P., Owen J., Abraham J., Barrett S., Barrett-Lee P. (2018). Carboplatin in BRCA1/2-mutated and triple-negative breast cancer BRCAness subgroups: The TNT Trial. Nat. Med..

[124] Robson M., Im S.-A., Senkus E., Xu B., Domchek S.M., Masuda N., Delaloge S., Li W., Tung N., Armstrong A. (2017). Olaparib for Metastatic Breast Cancer in Patients with a Germline BRCA Mutation. N. Engl. J. Med..

[125] Litton J.K., Rugo H.S., Ettl J., Hurvitz S.A., Gonçalves A., Lee K.-H., Fehrenbacher L., Yerushalmi R., Mina L.A., Martin M. (2018). Talazoparib in Patients with Advanced Breast Cancer and a Germline BRCA Mutation. N. Engl. J. Med..

[126] Rugo H.S., Ettl J., Hurvitz S.A., Gonçalves A., Lee K.-H., Fehrenbacher L., Mina L.A., Diab S., Woodward N.E., Yerushalmi R. (2020). Outcomes in Clinically Relevant Patient Subgroups from the EMBRACA Study: Talazoparib vs Physician’s Choice Standard-of-Care Chemotherapy. JNCI Cancer Spectr..

[127] Litton J.K., Hurvitz S.A., Mina L.A., Rugo H.S., Lee K.-H., Gonçalves A., Diab S., Woodward N., Goodwin A., Yerushalmi R. (2020). Abstract CT071: Talazoparib (TALA) in Germline BRCA1/2 (gBRCA1/2)-Mutated Human Epidermal Growth Factor Receptor 2 Negative (HER2-) Advanced Breast Cancer (ABC): Final Overall Survival (OS) Results from Randomized Phase 3 EMBRACA Trial.

[128] Han H.S., Diéras V., Robson M., Palácová M., Marcom P.K., Jager A., Bondarenko I., Citrin D., Campone M., Telli M.L. (2018). Veliparib with temozolomide or carboplatin/paclitaxel versus placebo with carboplatin/paclitaxel in patients with BRCA1/2 locally recurrent/metastatic breast cancer: Randomized phase II study. Ann. Oncol..

[129] Zeichner S.B., Terawaki H., Gogineni K. (2016). A Review of Systemic Treatment in Metastatic Triple-Negative Breast Cancer. Breast Cancer Basic Clin. Res..

[130] Cardoso F., Senkus E., Costa A., Papadopoulos E., Aapro M., André F., Harbeck N., Lopez B.A., Barrios C., Bergh J. (2018). 4th ESO–ESMO international consensus guidelines for advanced breast cancer (ABC 4). Ann. Oncol..

[131] Twelves C., Cortes J., Vahdat L., Olivo M., He Y., Kaufman P.A., Awada A. (2014). Efficacy of eribulin in women with metastatic breast cancer: A pooled analysis of two phase 3 studies. Breast Cancer Res. Treat..

[132] Bardia A., Tolaney S.M., Loirat D., Punie K., Oliveira M., Rugo H.S., Brufsky A., Kalinsky K., Cortés J., O’Shaughnessy J. (2020). LBA17 ASCENT: A randomized phase III study of sacituzumab govitecan (SG) vs treatment of physician’s choice (TPC) in patients (pts) with previously treated metastatic triple-negative breast cancer (mTNBC). Ann. Oncol..

